# Comparative genomic analysis of Polypodiaceae chloroplasts reveals fine structural features and dynamic insertion sequences

**DOI:** 10.1186/s12870-020-02800-x

**Published:** 2021-01-07

**Authors:** Shanshan Liu, Zhen Wang, Yingjuan Su, Ting Wang

**Affiliations:** 1 School of Life Sciences, Sun Yat-sen University, Guangzhou, Guangdong China; 2 Research Institute of Sun Yat-sen University in Shenzhen, Shenzhen, Guangdong China; 3 College of Life Sciences, South China Agricultural University, Guangzhou, Guangdong China

**Keywords:** Plastome, Polypodiaceae, Insertions, Dynamic evolution

## Abstract

**Background:**

Comparative chloroplast genomics could shed light on the major evolutionary events that established plastomic diversity among closely related species. The Polypodiaceae family is one of the most species-rich and underexplored groups of extant ferns. It is generally recognized that the plastomes of Polypodiaceae are highly notable in terms of their organizational stability. Hence, no research has yet been conducted on genomic structural variation in the Polypodiaceae.

**Results:**

The complete plastome sequences of *Neolepisorus fortunei*, *Neolepisorus ovatus*, and *Phymatosorus cuspidatus* were determined based on next-generation sequencing. Together with published plastomes, a comparative analysis of the fine structure of Polypodiaceae plastomes was carried out. The results indicated that the plastomes of Polypodiaceae are not as conservative as previously assumed. The size of the plastomes varies greatly in the Polypodiaceae, and the large insertion fragments present in the genome could be the main factor affecting the genome length. The plastome of *Selliguea yakushimensis* exhibits prominent features including not only a large-scale IR expansion exceeding several kb but also a unique inversion. Furthermore, gene contents, SSRs, dispersed repeats, and mutational hotspot regions were identified in the plastomes of the Polypodiaceae. Although dispersed repeats are not abundant in the plastomes of Polypodiaceae, we found that the large insertions that occur in different species are mobile and are always adjacent to repeated hotspot regions.

**Conclusions:**

Our results reveal that the plastomes of Polypodiaceae are dynamic molecules, rather than constituting static genomes as previously thought. The dispersed repeats flanking insertion sequences contribute to the repair mechanism induced by double-strand breaks and are probably a major driver of structural evolution in the plastomes of Polypodiaceae.

## Background

Chloroplasts are the plant organelles in which photosynthesis takes place. Each chloroplast contains its own genome (plastome), which usually occurs in multiple copies within the organelle [1]. In recent years, the chloroplast (cp) genome has become a preferred target for comparative genomics because of its mostly uniparental inheritance, compact size, lack of recombination, and moderate evolutionary rate compared to the two other genomes found in plant cells (nuclear and mitochondrial genomes) [2–4]. Advances in DNA sequencing technology have provided highly efficient, cost-effective sequencing platforms, and the properties of the plastome made it one of the first candidates for high-throughput sequencing and assembly. Plastomes have now been extensively used for exploring phylogenetic relationships and understanding evolutionary processes of plants [5–7].

Ferns, which are the second largest group of vascular plants, play important ecological roles and hold a pivotal phylogenetic position [8]. The sequencing of fern plastomes has greatly increased our understanding of the plastomic diversity and evolution of this lineage. The sizes of the plastomes of ferns are highly conserved, and they usually exhibit a circular structure ranging from 120 to 170 kb [9]. The plastomes of ferns typically consist of four parts, including a pair of large inverted repeats (IRs), a large single copy (LSC) region, and a small single copy (SSC) region. Almost all fern IRs contains a core gene set of four ribosomal RNAs (16S, 23S, 4.5S, and 5S) and several tRNA genes (*trn*A-UGC, *trn*I-GAU, *trn*N-GUU, and *trn*R-ACG). Structurally, the plastome of fern lineages has evolved in a conservative manner. Most fern plastomes are largely collinear, requiring only a few inversions and IR expansions to account for the large-scale structural rearrangements observed among major lineages. For example, the unique chloroplast genomic rearrangement of core leptosporangiate ferns (Salviniales, Cyatheales, and Polypodiales) and Schizaeales can be explained by an expansion of the IRs and “two inversions” [10], which mainly affect the orientation and gene content of the IRs [11]. The conservative nature of the plastome makes it homogeneous enough to allow comparative studies to be conducted across higher-level taxa, but it is also sufficiently divergent to capture various evolutionary events.

The Polypodiaceae family is one of the most species-rich groups of extant ferns [12], displaying remarkable morphological and systematic diversity. Leptosporangiate ferns diversified in an angiosperm-dominated canopy during the Cenozoic radiation period, thus establishing the diversity of the Polypodiaceae [13]. Most species within Polypodiaceae are epiphytes, and this family represents one of the most diverse and abundant groups of pantropical vascular epiphytes in tropical and subtropical forests [14, 15]. The plastomes of Polypodiaceae have undergone a variety of complex genomic reconstructions over evolutionary time, making them significantly different from those of the basal ferns (Marattiales, Ophioglossales, Psilotales, and Equisetales). Few studies have analyzed the gene content and structural changes of Polypodiaceae plastomes in detail because plastome evolution in Polypodiaceae is considered relatively dormant compared with that in other lineages. Most of the relevant research has focused on phylogenetic topics. Recent studies, however, have shown that the plastomes of polypods contain not only hypervariable regions but also widespread mobile sequences [16, 17]. Unfortunately, the corresponding studies have rarely involved Polypodiaceae because of the limited available plastome data for this group. This indicates that the currently available plastome information may be insufficient to elucidate the evolutionary patterns of the fern genome, and the exploration of the structural diversity of Polypodiaceae plastomes is also far from sufficient. Comparative genomic analyses of the chloroplasts among closely related species can generate genetic markers and provide a more exhaustive understanding of the evolutionary trajectory of the genome [18, 19]. Nevertheless, the plastome structural and sequence homogeneity among low-level taxa makes it difficult to identify various evolutionary events. Therefore, it is necessary to compare the fine structural characteristics of Polypodiaceae plastomes to increase the understanding of the diversity and dynamic evolution of fern plastomes. Upon this premise, we performed the first family-scale comparative analysis of plastome structure and content in Polypodiaceae.

We utilized a high-throughput sequencing platform to assemble three new plastomes, two from *Neolepisorus* and one from *Phymatosorus*, and used them to perform detailed comparative analysis with the set of all previously published Polypodiaceae plastomes in an effort to: 1) characterize the genomic structure and gene content of newly sequenced plastomes; 2) examine Polypodiaceae plastome variation at the fine structural level; and 3) gain insight into the plastome evolution of Polypodiaceae.

## Results

### Genome assembly and annotation

**Fig. 1:**
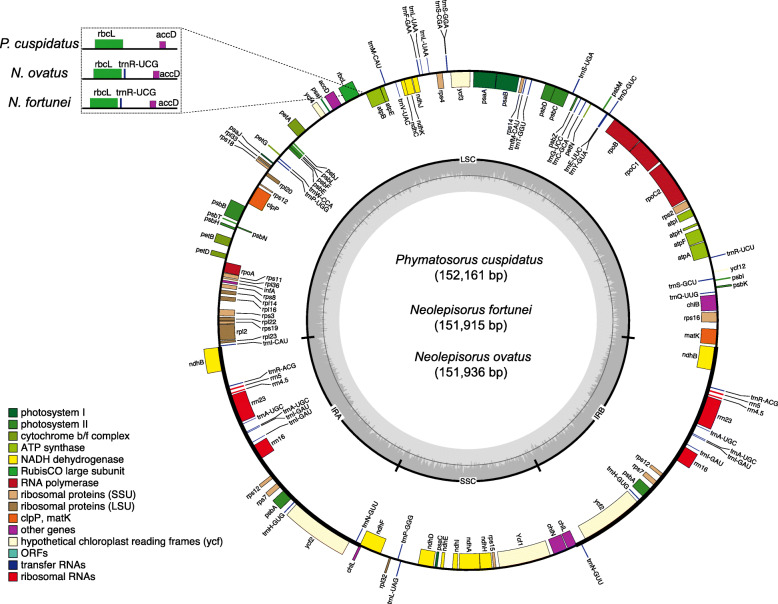
Plastome gene maps of *N. ovatus*, *N. fortunei*, and *P. cuspidatus*. The plastome map represents all three species since their gene numbers, orders and names are the same, except that *N. fortunei* has lost the *trnR*-UCG gene. Genes located outside and within the black circle are transcribed in the clockwise and counterclockwise directions, respectively. Different colors represent genes belonging to different functional groups

Illumina paired-end sequencing generated 6,760,171, 6,821,122, and 6,764,140 raw reads from *Neolepisorus fortunei*, *Neolepisorus ovatus*, and *Phymatosorus cuspidatus*, respectively (Table 1). A total of 635,763 to 1,054,231 clean reads were mapped to the reference plastome and 11 to 16 contigs were assembled for three species, reaching over 160× coverage on average over the plastomes. The draft plastome of *N. fortunei*, *N. ovatus*, and *P. cuspidatus* had six, two, and three gaps, respectively. The gap regions for each resulting plastome were filled by using PCR-based sequencing with corresponding pairs of primers (Table S1). The length of the complete plastome sequences ranged from 151,915 to 152,161 bp, with an average GC content of 41.7% (range 41.3–42.3%; Table 1). All plastomes exhibited the typical quadripartite structure, harboring a pair of large IRs (24,609–24,756 bp). The two IR regions divide the plastomes into an LSC region (80,670–81,175 bp) and an SSC region (21,601–21,733 bp) (Fig. 1, Table 2). The three newly sequenced plastomes have a similar gene content, with a few notable distinctions. Compared to the other two plastomes, loss of *trnR*-*UCG* is observed in *P. cuspidatus* (Fig. 1). *rps16* harbors an approximately 470-bp intronic deletion in *N. fortunei* (Fig. S1). Furthermore, *N. fortunei* has extra complete copies of *ndhB* in IRb because of its IRa/LSC border adjacent to *ndh*B, whereas *N. ovatus* and *P. cuspidatus* only contains a second fragment of *ndhB* in IRb due to their IRa/LSC border lies within *ndh*B.

**Table 1:** Details of plastome sequencing and assembly

Sample	*N. ovatus*	*N. fortunei*	*P. cuspidatus*
Raw Reads	6,760,171	6,821,122	6,764,140
Clean reads	5,765,899	5,907,868	5,880,444
Raw data	2.03G	2.05G	2.03G
Clean data	1.73G	1.77G	1.76G
Contig	13	11	16
Genome coverage (×)	313	292	160
Size (bp)/GC%	151,936/42.3	151,915/41.3	152,161/41.4

**Table 2:** Comparison of general features of the plastomes of Polypodiaceae

Subfamily	Microsoroideae								Platycerioideae		Drynarioideae	
Species	*NO*	*NF*	*PC*	*PN*	*LC*	*LHem*	*LM*	*LHed*	*PBi*	*PBo*	*DR*	*SY*
LSC (bp)	81,117	80,670	81,175	81,506	81,093	81,403	81,244	81,395	79,002	82,479	86,040	80,975
SSC (bp)	21,601	21,733	21,688	21,759	21,679	25,492	21,797	21,756	21,485	21,723	24,433	19,848
IR (bp)	24,609	24,756	24,649	24,643	27,113	24,594	27,494	24,593	28,249	26,986	23,416	32,017
Size (bp)	151,936	151,915	152,161	152,551	156,998	156,083	158,029	152,337	156,985	158,174	154,305	164,857
PGGs	88	89	88	88	88	87	88	88	88	88	88	91
tRNAs	35	35	34	35	35	34	35	35	33	35	35	35
rRNA	8	8	8	8	8	8	8	8	8	8	8	8
Pseudo	1	0	1	1	0	2	1	1	1	1	1	1
Genes	132	132	131	132	131	131	132	132	130	132	132	135
LSC (GC%)	40.2	42.1	40.4	41.5	40.46	43.6	40.5	41.9	37.9	40.1	39.8	39.5
SSC (GC%)	37.5	38.8	37.6	39.0	37.9	43.0	37.9	39.7	34.4	37.4	36.6	39.8
IR (GC %)	44.9	45.1	44.8	45.3	45.4	45.9	45.3	45.2	44.9	45.6	45.1	42.7
GC (Total%)	41.3	42.3	41.4	42.4	41.8	44.2	41.8	42.7	39.9	41.6	40.9	40.8

*NO* N. ovatus, *NF* N. fortunei, *PC* P. cuspidatus, *PN* P. niponica, *LC* L. clathratus, *LHem* L. hemionitideus, *LM* L. microphyllum, *LHed* L. hederaceum, *PBi* P. bifurcatum, *PBo* P. bonii, *DR* D. roosii, *SY* S. yakushimensis*,* *PCG* protein-coding gene, *Pseudo* pseudogene

### Whole-chloroplast genome comparison among Polypodiaceae

The three newly obtained plastomes of Polypodiaceae (*N. ovatus*, *N. fortunei*, and *P. cuspidatus*) were compared with nine previously published plastomes representing three subfamilies of Polypodiaceae, i.e., Microsoroideae, Platycerioideae, and Drynarioideae (Table 2). The Polypodiaceae plastomes appeared to be structurally similar to each other, showing a typical quadripartite structure consisting of two IRs separated by LSC and SSC. Overall, the analysis showed that the size of the plastome varies widely among Polypodiaceae, ranging from 151,936 bp in *N. ovatus* to 164,857 bp in *Selliguea yakushimensis*. The lengths of the LSC and SSC regions of most species are approximately 81 kb and 21 kb, respectively, but these two regions of the *Drynaria roosii* plastome are significantly larger, reaching 86 kb and 24 kb, respectively. Furthermore, the plastome of *Leptochilus hemionitideus* also contains a larger SSC (25,492 bp). The IR regions of the Polypodiaceae plastomes show significant variation in length compared to the SC regions, varying from 23,416 to 32,017 bp. The base composition of the Polypodiaceae plastomes is more conservative relative to their size variation. The distribution of GC content is heterogeneous, with the highest being observed in IR regions (42.1–45.9%), followed by LSC (37.9–43.6%), while the lowest is found in the SSC region (34.4–43.0%) (Table 2).

**Fig. 2:**
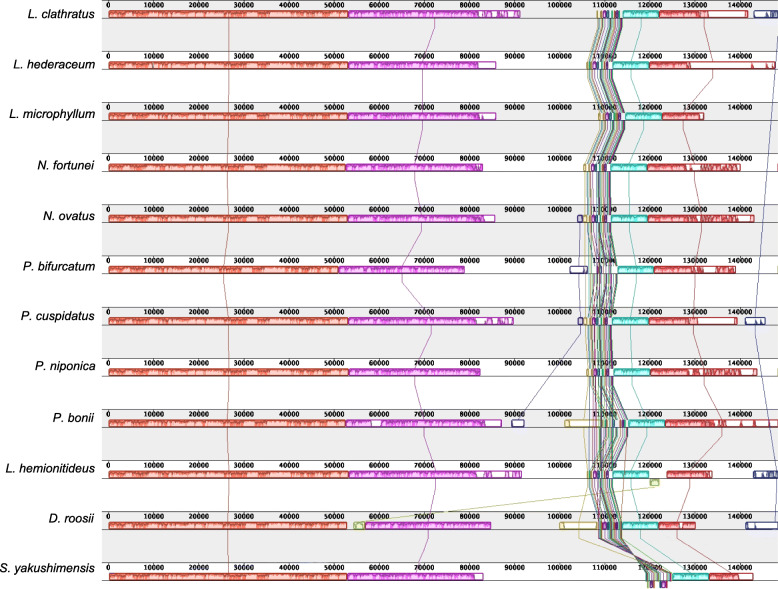
Synteny and rearrangements detected in the plastome sequences of Polypodiaceae using Mauve alignment. Local collinear blocks are represented as boxes of the same color connected with lines, which indicate syntenic regions. Histograms within each block represent the sequence identity similarity profile. The colored blocks, which are above and below the center line, represent sequences transcribed in reverse directions

The gene order of the plastomes of Polypodiaceae is almost collinear. However, the structure of the *S. yakushimensis* plastome is considerably different from the typical structure of Polypodiaceae. A notable difference in the plastome of *S. yakushimensis* is a transposition of an ~ 6 kb segment spanning *ndh*F to *ccs*A from the SSC to the IR; this inversion appears at the junction of IRb/SSC (Fig. 2). At another junction, SSC/IRa, we observed an extensive IR expansion resulting in the duplication of *ycf*1, *chl*L, and *chl*N at the end of IRa. In addition, a minor inversion (~ 2 kb) was detected between *Lepidomicrosorum hederaceum* and *D. roosii* plastomes, which was located in *rps*15-*ycf*1 in the SSC region of *L. hederaceum* and *rrn*L-*trn*R in the LSC region of *D. roosii*, respectively (Fig. 2). Genomic structural changes that occur in intergenic regions may play an additional evolutionary role, but they are difficult to detect because intergenic regions have no coding function. This unique inversion may be related to inserted sequenced or is an intermediate form of the evolution of other plastomes. As the number of published Polypodiaceae plastomes increased, the evolutionary processes of Polypodiaceae should become clearer.

**Fig. 3:**
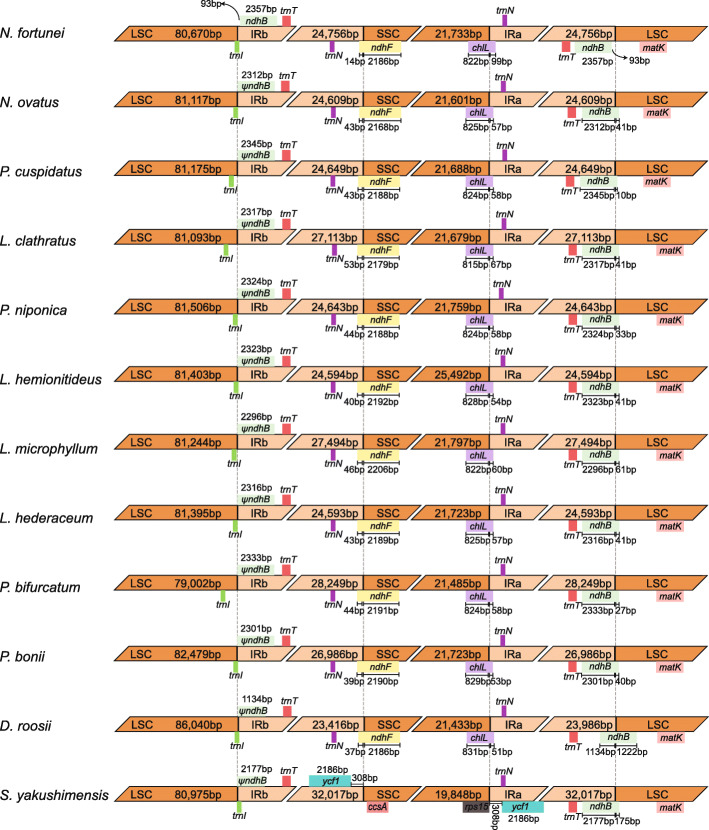
Comparison of the border positions of LSC, SSC, and IR regions among the plastomes of Polypodiaceae species. ψ refers to the pseudogene of *ndh*B at the LCS/IRb border

The size variations of the plastome may be a result of the dynamic changes of IR boundaries [20]. However, the Polypodiaceae plastomes exhibit high similarity at SC/IR boundaries, except in *S. yakushimensis* (Fig. 3). The *trnN-*GUU gene is located in the IR adjacent to either *ndhF* or *chlL* at the SSC/IR border. The *ndhF* and *chlL* genes cross the SSC/IR border, extending from 14 to 53 bp and 51–99 bp in the IR, respectively. The IRa/LSC border is located within the coding region of *ndh*B and generates a pseudogene of 1,134–2,345 bp at the LSC/IRb border in 11 of the plastomes, except for that of *N. fortunei*. The LSC/IRb border is located within or next to the *trnI*-CAU gene. In contrast to the subtle changes in IR boundaries, the IR of *S. yakushimensis* has experienced extensive expansion, capturing the *ycf*1, *chl*L, and *chl*N genes in the SSC region. This expansion, combined with the inversion of the *ndh*F-*ccs*A region, causes a unique SSC/IR boundary in the *S. yakushimensis* plastome (Fig. 3). The SC/IR boundaries of the Polypodiaceae plastomes are highly similar but not identical, indicating that the expansion and contraction of IR is an independent and recurrent phenomenon in evolution. Furthermore, the change in IR boundaries is not sufficient to cause a large difference in genome size, and we believe that microstructural changes (such as insertions and deletions) may be responsible for the difference.

There are some variations in the gene content of Polypodiaceae plastomes due to varying degrees of IR boundary changes and inversions. *trn*R-UCG exists in all Polypodiaceae species except for *L. hemionitideus*, *P. cuspidatus*, and *Platycerium bifurcatum*. *trn*V-UAC is also absent in the plastome of *P. bifurcatum*. Another difference is that the plastome of *N. fortunei* contains an additional intact *ndhB* gene in the IR region, whereas only one *ndhB* fragment exists in other species. Furthermore, close inspection of the gene annotations of the 12 Polypodiaceae plastomes indicated that the *rpoC1* gene of the *L. hemionitideus* has been pseudogenized by a frameshift mutation.

### Sequence diversity and mutational hotspots of the Polypodiaceae plastome

**Fig. 4:**
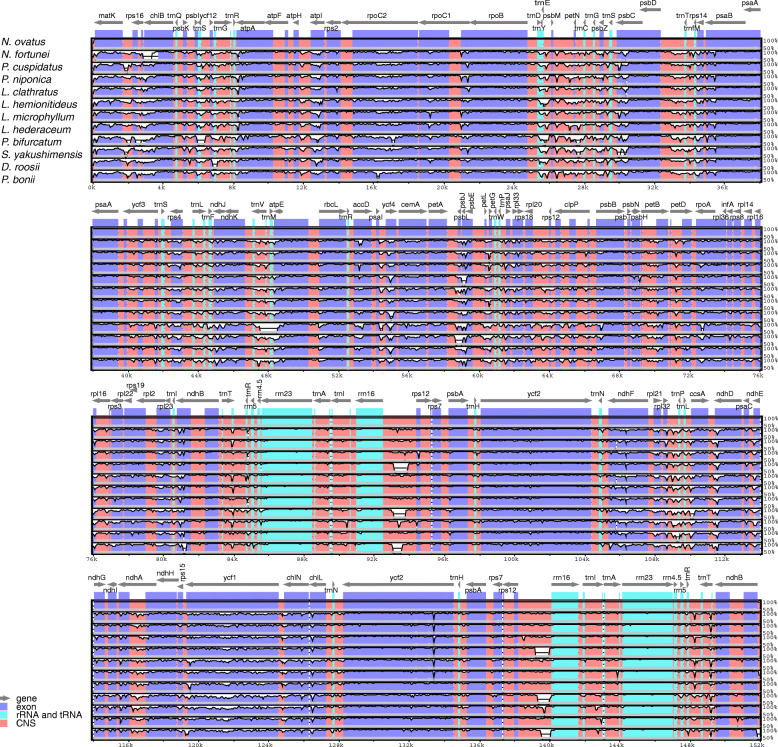
Visualization of the alignment of the Polypodiaceae plastome sequences, with *N. fortunei* as the reference. Gray arrows above the alignment indicate genes, including their orientation and position. The vertical scale represents the percentage of identity, ranging from 50 to 100%. Genome regions are color-coded as protein-coding genes, tRNAs, rRNAs and conserved noncoding sequences (CNSs)

The multiple sequence alignments performed in mVISTA software showed the similarity of the whole sequences of the plastomes of the 12 Polypodiaceae species analyzed (Fig. 4). Lower divergence was found in the IR and protein-coding regions than in the single-copy and noncoding regions. Nevertheless, we found that obvious large inserted fragments were present in the *rrn*16-*rps*12 spacer of the IRs in the plastomes of *Lepisorus clathratus*, *Pyrrosia bonii*, and *P. bifurcatum*. To detect mutational hotspots in the plastomes of Polypodiaceae, sliding-window analysis was performed on the whole-genome alignments of the sequences using DnaSP v5.0. The results showed that the sequence variation between the plastomes of Polypodiaceae was relatively low, with nucleotide diversity (Pi) ranging from 0.00232 to 0.20028. Overall, the SSC region exhibited the highest sequence variation, with an average Pi of 0.10103, followed by the LSC and IR regions, with average Pi values of 0.07317 and 0.02676, respectively. A total of nine highly divergent loci were identified in the plastomes of Polypodiaceae, including *mat*K-*rps*16, *rps*16, *trn*C-*trn*G, *psb*Z-psbC, *psb*D-*trn*T, *trn*P-*psa*J, and *rpl*2-*trn*I, located in LSC; *rrn*16-*rps*12, located in the IR region; and one protein-coding gene, *ycf*1, located in SSC. The *ndh*F-*ccs*A region in SSC showed a higher Pi value than the other loci, most likely due to the inversion occurring in this region, which resulted in higher sequence variation (Fig. S2). Therefore, it may not be categorized as a common mutational hotspot in the Polypodiaceae.

### Analysis of SSRs and repeat sequence in Polypodiaceae plastomes

**Fig. 5:**
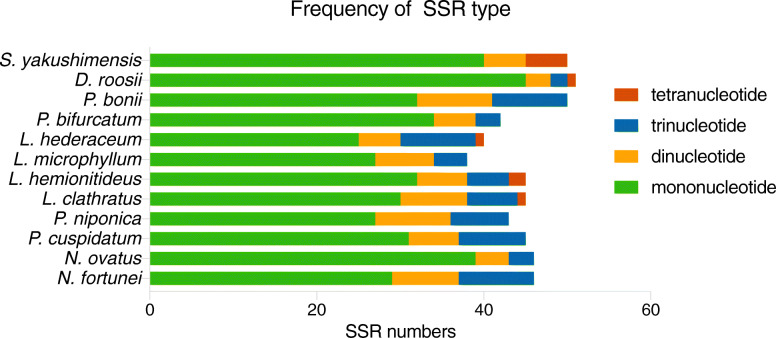
Types and numbers of cpSSRs in the Polypodiaceae plastomes

Simple sequence repeats (SSRs), or microsatellites are short, tandemly repeated DNA motifs of 1–6 nucleotides [21]. They can exhibit high polymorphism and mutation rates, which contribute to estimates of genetic variation [22]. In this study, very similar numbers of potential cpSSRs were identified from the plastomes of the 12 Polypodiaceae species by using MISA. The total number of SSRs in Polypodiaceae ranged from 38 to 51. Four kinds of SSRs were detected: mononucleotides, dinucleotides, trinucleotides, and tetranucleotides (Fig. 5). However, tetranucleotide repeats were discovered in only the plastomes of *L. clathratus*, *L. hemionitideus*, *L. hederaceum*, *S. yakushimensis*, and *D. roosii*. Different SSR motifs appeared at different frequencies in these plastomes. The most abundant observed repeats were mononucleotides, accounting for approximately 62.8–88.3% of the total number of SSR loci, followed by smaller numbers of dinucleotide (8.7–20.9%) and trinucleotide (6.6–22.5%) repeats, whereas tetranucleotide repeats were the least common (0–4.4%).

**Fig. 6:**
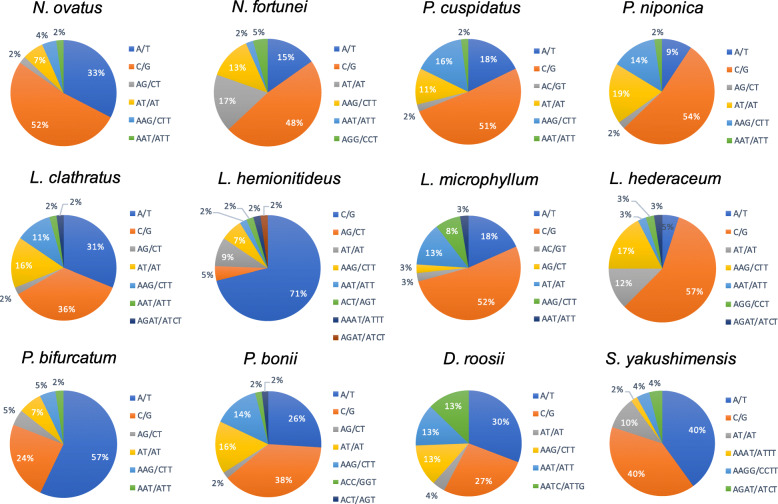
Frequency of identified SSR motifs in different repeat class types

We found that the predominant mononucleotide repeats in all analyzed species with the exception of *D. roosii*, *S. yakushimensis*, and *P. bifurcatum* were G/C tandem motifs, which accounted for 53.3 to 100% of the mononucleotide repeats in the Polypodiaceae plastomes (Fig. 6). The Polypodiaceae species included in this study all exhibited similar SSR distribution patterns in the plastome. SSRs were much more frequently located in the LSC region (48.0–71.1%) than in IR (10.5–36.0%) and SSC regions (9.3–22.0%). Furthermore, the majority of the identified SSRs were located in intergenic spacers, accounting for 66.7–83.3% of all SSRs detected. SSRs dispersed in intronic regions were the second most common category (9.5–28.9%). The fewest SSRs were located in coding genes, which accounted for only 4.0–14.0% of all SSR loci (Table S2).

Forward, palindromic and reverse repeats of more than 30 bp with a sequence identity ≥90% were detected in the Polypodiaceae plastomes using REPuter. The results showed that the numbers of repeats in the plastomes of Polypodiaceae varied considerably, ranging from 9 in *L. hemionitideus* to 146 in *P. bifurcatum*. These long repeats ranged from 30 to 307 bp in length and were repeated twice. Species showed some variation in the number of long repeat sequences located in intergenic spacers and coding genes. Most repeats were distributed in intergenic spacer regions, and the rest were distributed in coding genes and intronic regions (Table S3). We detected some species-specific intergenic spacers with rich repeats, including the *rbcL*-*trnR*-UCG intergenic spacer of the LSC region of *D. roosii*, the *rps*12-*rrn*16 intergenic spacer of the IR regions of *L. clathratus* and *P. bifurcatum*, the *rps7-psbA* intergenic spacer of the IR region of *Lemmaphyllum microphyllum*, and the *ndh*A intronic/*chl*L-*chl*N intergenic spacer of the SC/IR junction of *P. bonii*.

Tandem Repeats Finder v4.09 [23] was further used to identify the tandem repeats present in Polypodiaceae plastomes, with the minimum identity and size of the repeats set to 90% and 15 bp in unit length. A small number of tandem repeats were detected in all species except for *P. bifurcatum* and *L. hemionitideus* (Table 3). Among the species in which tandem repeats were detected, *L. microphyllum* exhibited the most repeats, and *P. bonii* exhibited the fewest. The intergenic spacers *rrn16*-*rps12*, *rps7-psbA*, and *rbcL*-*trnR*-UCG were regions containing abundant repeat sequences in the plastomes of *L. clathratus*, *L. microphyllum*, and *D. roosii*, respectively. All detected tandem repeats were distributed in noncoding regions, and the proportions of tandem repeats located in intergenic spacers were higher than those in intronic regions in Polypodiaceae species (Table 3).

**Table 3:** Size, number, and distribution of tandem repeats in the plastomes of Polypodiaceae

Sample	Motif	Count	Start-end	Location
*N. ovatus*	19	4	83,880-83,955	*trn*T-UGU intron
	23	2.3	108,602-108,647	*rpl*21-*rpl*32
	19	4	149,099-149,174	*trn*T-UGU intron
*N. fortunei*	26	2	27,472-27,523	*psb*M-*pet*N
	15	3	52,744-52,788	*trn*R-UCG-*acc*D
	15	3.9	116,723-116,781	*ndh*A-intron
*P. cuspidatus*	45	2	1719-1809	*mat*K-*rps*16
	26	2.5	2183-2248	*mat*K-*rps*16
	15	3	113,805-113,849	*psaC*-*ndhE*
*P. niponica*	18	3	25,927-25,980	*rpoB*-*trnD*-GUC
	15	5	53,389-53,463	*trnR*-UCG-*accD*
*L. clathratus*	24	2	1894-1941	*matK*-*rps16*
	22	2	21,463-21,506	*rpoC1* intron
	26	2	93,242-93,293	*rrn16*-*rps12*
	27	3	93,291-93,380	*rrn16*-*rps12*
	69	2	93,405-93,542	*rrn16*-*rps12*
	46	4	93,470-93,653	*rrn16*-*rps12*
	46	4	144,421-144,604	*rps12-rrn16*
	27	3	144,716-144,796	*rps12-rrn16*
	18	4	144,797-144,848	*rps12-rrn16*
*L. microphyllum*	30	1.9	12,813-12,870	*atp*H-*atp*I
	18	3.1	97,069-97,123	*rps*7-*psb*A
	47	2.2	98,673-98,773	*rps*7-*psb*A
	27	1.9	98,749-98,800	*rps*7-*psb*A
	27	2.9	98,870-98,948	*rps*7-*psb*A
	15	5	119,960-120,034	*ndh*A intron
	27	2.9	140,326-140,404	*psb*A- *rps*7
	27	1.9	140,474-140,525	*psb*A- *rps*7
	47	2.2	140,501-140,601	*psb*A- *rps*7
	18	3.1	142,151-142,205	*psb*A- *rps*7
*L. hemionitideus*	22	2	19,153-19,197	*rpo*C2-*rpo*C1
	25	1.9	74,525-74,572	*rps*11-*rpl*36
*P. bonii*	23	2	120,079-120,124	*ndhA* intron
*D. roosii*	146	2	5433-5711	*trnQ-*UGG*-psbK*
	23	2	19,077-19,122	*rpoC2-rpoC1*
	18	3	54,433–54,486	*rbcL*-*trnR*-UCG
	47	3	54,550-55,871	*rbcL*-*trnR*-UCG
	42	2	54,644-54,727	*rbcL*-*trnR*-UCG
*S. yakushimensis*	26	2	26,779-26,831	*psb*M-*pet*N
	28	3.2	125,129-125,218	*ndh*F-*ndh*D

### Dynamic insertion sequences in Polypodiaceae plastomes

Through a detailed whole-genome alignment, we found that there are large insertions in some regions of Polypodiaceae plastomes, including the *rrn*16-*rps*12 spacers of *L. clathratus*, *P. bifurcatum*, and *D. roosii* (~ 2400–3500 bp insertions); the *rps*7-*psb*A spacer of *L. microphyllum* (~ 3000 bp insertion); the *rbc*L-*trn*R spacer of *D. roosii* (~ 4000 bp insertion); the *pet*A-*psa*J spacer of *P. bonii* (~ 1700 bp insertion); and the *rps*15-*ycf*1 spacer of *L. hederaceum* (~ 3700 bp insertion) (Table 4). The identity of the insertion sequences in the *rrn*16-*rps*12 spacers of *L. clathratus*, *P. bifurcatum* and *D. roosii* was calculated using MegAlign v8.1.3 [24]. The pairwise alignments showed that the identity of the insertions in the three plastomes was only 48.3–50.1%, indicating that these insertions may have different origins (Fig. S3).

**Table 4:** Comparison of the length of intergenic regions containing large insertions in the plastomes of Polypodiaceae

Species	*rrn*16-*rps*12	*rps*7-*psb*A	*rbc*L-*trn*R	*pet*A*-psa*J	*rps*15*-ycf*1
*N. ovatus*	1896 bp	478 bp	128 bp	697 bp	311 bp
*N. fortunei*	1924 bp	475 bp	123 bp	682 bp	261 bp
*P. cuspidatus*	1902 bp	482 bp	130 bp	679 bp	313 bp
*P. niponica*	1916 bp	485 bp	123 bp	681 bp	311 bp
*L. clathratus*	**4420 bp**	479 bp	123 bp	464 bp	312 bp
*L. hemionitideus*	1903 bp	473 bp	126 bp	631 bp	**4069 bp**
*L. microphyllum*	1893 bp	**3403 bp**	127 bp	686 bp	300 bp
*L. hederaceum*	1893 bp	469 bp	123 bp	693 bp	308 bp
*P. bifurcatum*	**5476 bp**	479 bp	125 bp	717 bp	306 bp
*P. bonii*	**4362 bp**	470 bp	123 bp	**2475 bp**	304 bp
*D. roosii*	1902 bp	475 bp	**4107 bp**	669 bp	305 bp
*S. yakushimensis*	1377 bp	364 bp	126 bp	677 bp	309 bp

Regions with large insertions in different species are highlighted in bold.

Robison et al. [17] previously proposed the concept of MORFFO (Mobile Open Reading Frames in Fern Organelles), which are a set of mobile insertion sequences that are widely present in fern organelles. To verify whether the insertions detected in the Polypodiaceae plastomes are consistent with the MORFFO sequences, MORFFO sequences were determined by local BLAST searches [25, 26] using the database established from the 12 Polypodiaceae plastomes, with the consensus sequences of *morffo1*, *morffo2*, and *morffo3* as queries. Furthermore, to examine whether the insertions identified in this study present mobile properties, these sequences were subjected to local BLAST searches. Our results showed that *morffo1* presents similarity to the *pet*A-*psb*J fragment of *P. bonii* (71.2%) and the *rrn*16-*rps*12 fragment of *P. bifurcatum* (70.3%). *Morffo2* was detected in *rrn*16-*rps*12 of *P. bifurcatum* and *ycf*1-*ccs*A of *S. yakushimensis*, with identities of 71.0% and 67.9%, respectively (Table 5).

**Table 5:** Results of matches for insertion sequences within Polypodiaceae identified using local BLAST searches

Query	Subject	% Identity	Alignment length	Start	End	Location
*morffo1*	*P. bonii*	71.2	1217	59,496	58,295	*pet*A-*psb*J
	*P. bifurcatum*	70.3	1254	93,132	91,893	*rrn*16-*rps*12
*morffo2*	*P. bifurcatum*	71.0	1668	95,199	93,541	*rrn*16-*rps*12
	*S. yakushimensis*	67.9	1121	117,503	118,561	*ycf*1-*ccs*A
*L. clathratus* (*rrn*16-*rps*12)	*L. microphyllum*	91.5	1276	97,718	96,461	*rps*7-*psb*A
	*L.microphyllum*	88.1	796	98,773	97,983	*rps*7-*psb*A
	*D. roosii*	92.0	647	54,866	55,512	*rbc*L-*trn*R
	*D. roosii*	86.7	668	55,779	56,443	*rbc*L-*trn*R
	*L. hemionitideus*	81.4	737	121,798	121,063	*rps*15-*ycf*1
	*L. hemionitideus*	71.3	523	120,796	120,281	*rps*15-*ycf*1
*P. bifurcatum* (*rrn*16-*rps*12)	*P. bonii*	88.8	1502	58,225	59,724	*pet*A-*psb*J
	*P. bonii*	84.7	698	59,636	60,323	*pet*A-*psb*J
	*S. yakushimensis*	70.5	831	118,643	117,834	*ycf*1-*ccs*A
*L. microphyllum* (*rps*7-*psb*A)	*D. roosii*	87.0	1527	56,416	54,895	*rbc*L-*trnR*
	*L. clathratus*	91.5	1276	95,574	94,305	*rrn*16-*rps*12
	*L. clathratus*	88.1	796	94,304	93,538	*rrn*16-*rps*12
	*L. hemionitideus*	81.8	1425	120,281	121,697	*rps*15-*ycf*1
*D. roosii* (*rbc*L-*trn*R)	*L. microphyllum*	87.0	1527	98,595	97,078	*rps*7-*psb*A
	*L. hemionitideus*	79.3	1388	121,661	120,281	*rps*15-*ycf*1
	*L. clathratus*	92.0	647	93,658	94,304	*rrn*16-*rps*12
	*L. clathratus*	86.7	668	94,307	94,969	*rrn*16-*rps*12
*P. bonii* (*pet*A*-psa*J)	*P. bifurcatum*	88.8	1502	91,825	93,310	*rrn*16-*rps*12

Surprisingly, the insertions contained in different species show significant BLAST hits against each other, which suggests that DNA fragments may have been transferred from one plastome to another. For example, a fragment of the *L. clathratus* insertion shows high sequence similarity to the *rps*7-*psb*A insertion located in the IR of *L. microphyllum, rbc*L-*trn*R located in the LSC of *D. roosii*, and *rps*15-*ycf*1 located in the SSC of *L. hemionitideus*. *Morffo1* is located within the *P. bifurcatum*-*P. bonii* consensus insertion fragment, but *morffo2* does not show overlap with the insertions detected in this study (Table 5). Previous studies have revealed that MORFFO-like sequences are often associated with structural changes in the genome and may be the main driving force for structural evolution in the plastome. In this study, a number of movable insertions were found in the relatively static plastomes of Polypodiaceae, indicating that the different insertion fragments arising during the evolution of genome structure may have different functions.

## Discussion

### Plastome variation across Polypodiaceae

Our comparative analysis of 12 species from Microsoroideae, Platycerioideae, and Drynarioideae showed that the length of the plastomes varies greatly in Polypodiaceae, even within the same subfamily. Generally, dynamic expansions or contractions in IR boundaries are considered a primary mechanism leading to the size variation of land plant plastomes [20]. Although there are differences in the IR boundaries between Polypodiaceae plastomes, they also exhibit obvious similarities. Therefore, the minor shifts in the IR boundaries of the Polypodiaceae plastomes are expected to be insufficient to account for marked differences in genome size. For example, in Microsoroideae, we found that despite the IR boundaries being deeply conservative, the IR regions of *L. clathratus* and *L. microphyllum* are approximately 2500 bp longer than those of other species, and the SSC region of *L. hemionitideus* is approximately 3700 bp longer than those of other species. A fine-scale analysis of the plastome sequence data of Polypodiaceae revealed several large insertions in the specific intergenic spacers of IR or SC regions that correspond to the observed genome size differences. This situation corresponds well with those in the species of the other two subfamilies, with the exception of *S. yakushimensis*. Therefore, in most species of Polypodiaceae, the main reason for the difference in plastome size is not the variation in SC/IR boundaries but the large fragment insertions that occur in different species. *Selliguea yakushimensis* of Drynarioideae exhibits the largest IRs identified in the fern lineage to date, mainly because its IR boundary has extended toward the SSC, causing the *ycf*1, *chl*L, and *ch*lN genes to be captured within the IR region. Therefore, we cannot discuss the differences in the size of the plastomes only from the perspective of the expansion and contraction of the IR boundary because the conservative nature of the plastome itself will cause researchers to ignore other factors.

It is generally recognized that plastome evolution of Polypodiaceae has mostly stabilized, and structural changes such as rearrangements occur rarely. By contrast, the results presented here indicate that the plastome of *S. yakushimensis* is highly unusual in some respects, containing not only a large-scale IR expansion exceeding several kb but also a unique inversion. The large-scale expansion of the IR presumably occurred through double reciprocal recombination between IR segments during replication [27]. Numerous dispersed repetitive sequences located at the original SSC/IR junction (*chl*L/*trn*N-GUU) were detected in the *S. yakushimensis* plastome, coinciding exactly with the mechanism of IR expansion; that is, repeat sequences provide the potential for genome rearrangement within or between molecules by homologous recombination [27, 28]. In general, the homogeneity of plastome structural changes is low relative to the sequence data. The structural changes in the *S. yakushimensis* plastome can therefore serve as specific genetic markers in species discrimination or phylogenetic analyses. Unfortunately, there is no way to determine which event took place first because IR expansion has occurred at the SSC/IRa boundary, while inversion has occurred near the IRb/SSC boundary. Consequently, it is necessary to sequence more plastomes in Polypodiaceae to improve our understanding of the evolutionary trajectory of the plastome.

Whole-plastome alignments can elucidate the level of sequence divergence and easily identify large indels, which are extremely useful for phylogenetic analyses and plant identification. In the present study, our results showed that the IRs present lower sequence divergence than the SC regions. This phenomenon is considered to be a result of copy correction between IR sequences and the elimination of deleterious mutations by gene conversion [29]. Moreover, sequence differences among the Polypodiaceae plastomes were evident in the intergenic spacers, suggesting greater conservation in coding regions than in noncoding regions. Nine divergence hotspots between Polypodiaceae species were identified, including *mat*K-*rps*16, *rps*16, *trn*C-*trn*G, *psb*Z-psbC, *psb*D-*trn*T, *trn*P-*psa*J, *rpl*2-*trn*I, *rrn*16-*rps*12, and *ycf*1, among which nine loci were located in SC and one was located in an IR. Although the *ndh*F-*ccs*A regions also present higher Pi values, they may not be suitable as general mutational hotspots for Polypodiaceae due to the existence of inversion in this region of the *S. yakushimensis* plastome. Thus, we must conduct careful data exploration when screening universal mutational hotspots to avoid confusion by plastome structural changes.

In this study, we found many repeat regions, including forward repeats, palindromic repeats, and reverse and tandem structures, which could be important hotspots for genome reconfiguration [30, 31]. In particular, the occurrence of large repeats in plastomes, such as the 307 bp palindromic repeat observed in *P. cuspidatus,* has been speculated to result in an unstable genome structure due to inappropriate rearrangement [32]. In addition, these repeats provide many informative loci for the development of molecular markers for phylogenetics and population genetics [33]. As a very powerful type of molecular marker, SSRs are widely used in different research fields. They possess obvious advantages such as high polymorphism and cost effectiveness [34]. Most studies have shown that the predominant cpSSRs of land plants are consistent with their AT-biased plastomes. By contrast, the cpSSRs present in Polypodiaceae show considerable dissimilarity from the previously reported patterns. In this study, more than 38 SSRs were identified in every Polypodiaceae plastome, among which the majority were C/G mononucleotides and were distributed in noncoding regions. The previously held idea that cpSSRs are generally composed of A/T repeats has now been challenged [35]. Gao et al. [36] have shown in denaturation experiments that repetitive structures with a higher GC content contribute to increasing the thermal stability of the *Dryopteris fragrans* plastome and maintaining its structure in the face of thermal changes. Species of Polypodiaceae evolved diversified morphological traits and lifestyles putatively in response to changes in terrestrial ecosystems caused by the radiation of angiosperms during the Cretaceous period [37]. Thus, we speculate that these repeating structures with a high GC content may be one of the molecular foundations of the adaptation of Polypodiaceae to the environment, which also provides new insights for understanding the environmental adaptation mechanism of plants. Furthermore, the cpSSRs developed in our study provide unique information for investigating genetic structure and genetic variation. In particular, these cpSSRs will be complementary and comparable to nuclear SSRs from ferns.

### Dynamic insertions in Polypodiaceae plastomes

Although genome-wide alignment indicates that Polypodiaceae plastomes are rather conservative, we found abnormally large insertions in certain intergenic spacers. The large fragment insertion observed in the *rrn16*-*rps12* intergenic spacer of *L. clathratus* plastome is consistent with previous findings [16]. Similar insertion sequences have been detected in the LSC regions of some distantly related species and in the mitogenome of *Asplenium nidus*, implying that such sequences can move between genomic compartments [16]*.* Robison et al. [17] discovered a similar suite of dynamic mobile elements through an extensive investigation on fern plastomes, shedding light on the presence of MORFFO elements relative to inversions, intergenic expansions, and changes to inverted repeats. In this study, we characterized a completely different set of insertion sequences in the plastomes of Polypodiaceae. There are only two insertions that overlap with the *morffo1* sequence, which are located in the *pet*A-*psa*J spacer in the LSC region of *P. bonii* and the *rrn*16-*rps*12 spacer in the IR region of *P. bifurcatum*. The detected *morffo2* sequences are located in the *ycf*1-*ccs*A region at the IR/LSC border in *S. yakushimensis* and the *rrn*16-*rps*12 spacer in the IR region of *P. bifurcatum*, showing no homology with other insertions. Our results further confirm the universality of MORFFO sequences in fern plastomes, and the presence of such a sequence at the inversion endpoint in *S. yakushimensis* suggests that the MORFFO sequences may be related to inversion events.

Although MORFFO sequences were not detected in the remaining Polypodiaceae species, there is another set of highly mobile insertions present in their plastomes. It is worth noting that plastid genes are rarely gained or lost, whereas our study indicates that the identified insertion sequences have been gained and lost frequently during the evolution of Polypodiaceae plastomes. This fluidity could indicate that these insertions in plastomes act as mobile elements. Furthermore, these insertions are frequently found adjacent to regions where more dispersed repeats occur. Many studies have shown that genomic rearrangement is related to small dispersed repeats (SDRs), which contribute to the repair mechanism induced by double-strand breaks [38, 39]. SDRs usually make an important contribution to the repetitive space in highly rearranged genomes and increase structural polymorphism even in closely related lineages, and they are mainly present in noncoding DNA fragments and related to small hairpin structures [38]. We speculate that the presence of rich repetitive motifs combined with highly mobile insertions may constitute the “trigger mechanism” for genome rearrangement in the plastomes of Polypodiaceae species, which can induce structural changes in the plastome under certain conditions. The limitations of the current sequence data for ferns mean that it is difficult to determine the exact source of these insertion sequences. As more genomic data are published in the future, the source and migration mechanisms of these insertion sequences should become clear.

## Conclusions

As additional plastomes from Polypodiaceae are characterized, we obtain a clearer picture of plastome evolution for the family. It is generally considered that the evolution of Polypodiaceae plastomes is conservative, and their structural features are almost invariant in this family. Against this conservative background, however, the *S. yakushimensis* plastome stands out as unusual. The large-scale expansion of IRs and a unique inversion distinguish the *S. yakushimensis* plastome from those of all other Polypodiaceae studied thus far. In addition, many large mobile insertions are found in the plastomes of Polypodiaceae species, which often flank the dispersed repeated elements in the different Polypodiaceae plastomes. These unusual features are found in the structurally stable plastomes of Polypodiaceae and may therefore be implicated in the dynamic evolution of the plastomes of the family. In other words, unlike the static plastomes of Polypodiaceae characterized previously, the plastomes characterized herein are structurally unstable, as evidenced by the large mobile insertions found in Polypodiaceae.

## Methods

### Sample collection, DNA extraction, and sequencing

In this study, fresh leaves of *N. fortunei, N. ovatus,* and *P. cuspidatus* were sampled from the living collection from the South China Botanical Garden, Chinese Academy of Sciences (CAS), quickly frozen in liquid nitrogen, and stored at ultra-low- temperature refrigerator at − 80 °C until use. Voucher specimens were deposited in the Herbarium of Sun Yat-sen University (SYS; voucher: *S. Liu* 201,630, *S. Liu* 201,654, and *S. Liu* 201,701 for *N. fortunei, N. ovatus,* and *P. cuspidatus*, respectively). Genomic DNA was extracted using the Tiangen Plant Genomic DNA Kit according to the manufacturer’s instructions (Tiangen Biotech Co., Beijing, China). DNA quality was inspected in 0.8% agarose gels, and DNA quantification was performed using a NanoDrop spectrophotometer (Thermo Scientific, Carlsbad, CA, USA). After quality assessment, 500 ng of DNA was sheared to an average fragment size of 300 bp with a Covaris M220 ultrasonicator (Covaris Inc., MS, USA). An Illumina paired-end (PE) sequencing library was constructed using the NEBNext, Ultra DNA Library Prep Kit (New England BioLabs Inc., Ipswich, MA). Sequencing took place on the HiSeq 2500 platform (Illumina Inc., San Diego, USA). Illumina sequencing produced approximately 2 Gb of raw data for each species.

### Genome assembly and annotation

Raw reads were assessed for quality with FastQC v0.10.0 [40]. Low-quality bases (Q < 20) and adapter sequences were trimmed by using Trimmomatic v0.32 [41]. Clean reads were mapped against the reference plastome of *Lepisorus clathratus* (NC_035739) to filtered chloroplast data. All mapped reads were de novo assembled into contigs with Velvet [42], and were further aligned and oriented with the reference genome. Remaining gaps were filled by direct PCR using specific primers that were designed based on contig sequences or homologous sequence alignments. Chloroplast gene annotation was conducted using DOGMA [43], followed by manual correction of the start and stop codons and intron/exon boundaries based on homologous genes from other published closely related fern plastomes. Transfer RNA (tRNA) genes were verified using ARAGORN [44] and tRNAscan-SE in organellar search mode with default parameters [45]. A circular map of the plastome was drawn with OGDRAW [46]. The accession numbers of *N. fortunei, N. ovatus,* and *P. cuspidatus* were MT373087, MT364352, and MT364353, respectively.

### Comparative analysis of Polypodiaceae plastomes

The availability of multiple plastomes from Polypodiaceae provides an opportunity to explore the diversity of the genome within the family, including genome size and structure, GC content, gene order, and IR expansion/contraction. Therefore, we performed comparative analyses of three assembled plastome sequences and the nine other available plastomes of Polypodiaceae species, from *Polypodiodes niponica* (NC_040221), *Lepisorus clathratus* (NC_035739), *Leptochilus hemionitideus* (NC_040177), *Lemmaphyllum microphyllum* (MN623356), *Platycerium bifurcatum* (MN623367), *Lepidomicrosorum hederaceum* (MN623364), *Pyrrosia bonii* (NC_040226), *Selliguea yakushimensis* (MN623352), and *Drynaria roosii* (KY075853). The plastome of *D. roosii* was reannotated using gene prediction tools and manual adjustments before our analyses because of errors that we noticed in the *D. roosii* annotations. The genome size, gene content, IR boundaries, and base composition were compared based on the sequence and annotation information of these plastomes. Whole-genome alignments among the 12 Polypodiaceae species were performed to identify inversions using the ProgressiveMauve algorithm in Mauve v2.4.0 [47] after one inverted repeat (IR) copy was removed from each plastome.

The overall similarities of the 12 Polypodiaceae plastomes were plotted using the mVISTA [48] online program in Shuffle-LAGAN mode with the annotations of *N. fortunei* as a reference. To estimate nucleotide diversity (Pi) and mutational hotspots among Polypodiaceae species, we performed pairwise alignments of 12 plastomes in MAFFT v7.310 software [49], adjusted manually with BioEdit software [50] if necessary. The nucleotide diversity values (Pi) of the aligned sequences were calculated via sliding window analysis by using DnaSP v5.0 [51] with window lengths and step sizes of 600 and 200 bp, respectively.

### Characterization of SSRs and repeat sequences

Further comparisons between Polypodiaceae species were performed with the repetitive elements found in their chloroplast sequences. Simple sequence repeats (SSRs) were detected using the Perl script MISA [52] (MIcroSAtellite; http://pgrc.ipk-gatersleben.de/misa/), with minimal iterations of ten repeat motifs for mononucleotides, five for dinucleotide repeats, and four for tri-, tetra-, penta- and hexa-nucleotides. Tandem repeats in eight Polypodiaceae species were recognized using Tandem Repeats Finder v4.09 [23], with matches, mismatches, and indels set at 2, 7, and 7, respectively. The parameter settings were 90 for the minimum alignment score and 500 for the maximum period size. REPuter [53] (http://bibiserv.techfak.uni-bielefeld.de/reputer/) was used to visualize the location and size of the dispersed repeats (forward, reverse, complementary, and palindromic repeat sequences) with a minimal repeat size of 30 bp and a hamming distance of 3.

## Supplementary Information

**Additional file 1: Table S1.** Primers used for plastomes gap closing. **Table S2.** Distribution of SSRs in different plastome regions of Polypodiaceae. **Table S3.** Information on dispersed repeats among Polypodiaceae plastomes.

**Additional file 2: Figure S1.** Alignment of *rps*16 exons/introns for the three plastomes that we sequenced. The intron is deleted only in *N. fortunei*.

**Additional file 3: Figure S2.** Nucleotide diversity (Pi) in the plastomes of 12 Polypodiaceae species.

**Additional file 4: Figure S3.** Identity of large insert fragments in *rrn*16-*rps*12 among the plastomes of *L. clathratus, P. bifurcatum*, and *D. roosii*.

## Data Availability

Annotated sequences of plastomes of *N. fortunei, N. ovatus,* and *P. cuspidatus* were submitted to GenBank (https://www.ncbi.nlm.nih.gov/genbank/) under MT373087, MT364352, and MT364353 accession number, respectively. All data generated or analyzed during this study are included in the article and its additional files.

## Abbreviations

cp: Chloroplast
IR: Inverted repeat
rRNA: Ribosomal RNA
SSC: Small single copy
tRNA: Transfer RNA
LSC: Large single copy
SSR: Simple sequence repeat
SDR: Small dispersed repeat
MORFFO: Mobile Open Reading Frames in Fern Organelles

## References

[1] Douglas SE (1998). Plastid evolution: origins, diversity, trends. Curr Opin Genet Dev.

[2] Wolfe KH, Li WH, Sharp PM (1987). Rates of nucleotide substitution vary greatly among plant mitochondrial, chloroplast, and nuclear DNAs. Proc Natl Acad Sci U S A.

[3] Drouin G, Daoud H, Xia J (2008). Relative rates of synonymous substitutions in the mitochondrial, chloroplast and nuclear genomes of seed plants. Mol Phylogenet Evol.

[4] Smith DR (2015). Mutation rates in plastid genomes: they are lower than you might think. Genome Biol Evol.

[5] Palmer JD, Soltis DE, Chase MW (2004). The plant tree of life: an overview and some points of view. Am J Bot.

[6] Lehtonen S (2011). Towards resolving the complete fern tree of life. PLoS One.

[7] Chen ZD, Yang T, Lin L, Lu LM, Li HL, Sun M (2016). Tree of life for the genera of Chinese vascular plants. J Syst Evol.

[8] Page CN, Dyer AF (1979). The diversity of ferns, an ecological perspective. The experimental biology of ferns.

[9] Mower JP, Vickrey TL (2018). Structural diversity among plastid genomes of land plants. Adv Bot Res.

[10] Wolf PG, Rowe CA, Sinclair RB, Hasebe M (2003). Complete nucleotide sequence of the chloroplast genome from a leptosporangiate fern, *Adiantum capillus-veneris* L. DNA Res.

[11] Hasebe M, Iwatsuki K (1992). Gene localization on the chloroplast DNA of the maiden hair fern; *Adiantum capillus-veneris*. Bot Mag Tokyo.

[12] PPG I (2016). A community-derived classification for extant lycophytes and ferns. J Syst Evol.

[13] Schuettpelz E, Pryer KM (2009). Evidence for a Cenozoic radiation of ferns in an angiosperm-dominated canopy. Proc Natl Acad Sci U S A.

[14] Gentry AH, Dodson C (1987). Diversity and biogeography of neotropical vascular epiphytes. Ann Mo Bot Gard.

[15] Benzing DH, Lowman MD, Rinker BH (2004). Vascular epiphytes. Forest canopies.

[16] Logacheva MD, Krinitsina AA, Belenikin MS, Khafizov K, Konorov EA, Kuptsov SV (2017). Comparative analysis of inverted repeats of polypod fern (Polypodiales) plastomes reveals two hypervariable regions. BMC Plant Biol.

[17] Robison TA, Grusz AL, Wolf PG, Mower JP, Fauskee BD, Sosa K (2018). Mobile elements shape plastome evolution in ferns. Genome Biol Evol.

[18] Shaw J, Lickey EB, Beck JT, Farmer SB, Liu W, Miller J (2005). The tortoise and the hare II: relative utility of 21 noncoding chloroplast DNA sequences for phylogenetic analysis. Am J Bot.

[19] Shaw J, Lickey EB, Schilling EE, Small RL (2007). Comparison of whole chloroplast genome sequences to choose noncoding regions for phylogenetic studies in angiosperms: the tortoise and the hare III. Am J Bot.

[20] Kim KJ, Lee HL (2004). Complete chloroplast genome sequences from Korean ginseng (*Panax schinseng* Nees) and comparative analysis of sequence evolution among 17 vascular plants. DNA Res.

[21] Li YC, Korol AB, Fahima T, Beiles A, Nevo E (2002). Microsatellites: genomic distribution, putative functions and mutational mechanisms: a review. Mol Ecol.

[22] Guichoux E, Lagache L, Wagner S, Chaumeil P, Léger P, Lepais O (2011). Current trends in microsatellite genotyping. Mol Ecol Resour.

[23] Benson G (1999). Tandem repeats finder: a program to analyze DNA sequences. Nucleic Acids Res.

[24] Burland TG, Misener S, Krawetz SA (2000). DNASTAR’s Lasergene sequence analysis software. Bioinformatics methods and protocols.

[25] Altschul SF, Gish W, Miller W, Myers EW, Lipman DJ (1990). Basic local alignment search tool. J Mol Biol.

[26] Madden T. The BLAST sequence analysis tool. 2nd ed: National Center for Biotechnology Information (US); 2013.

[27] Yamada T (1991). Repetitive sequence-mediated rearragements in *Chlorella ellipsoidea* chloroplast DNA: completion of nucleotide sequence of the large inverted repeat. Curr Genet.

[28] Palmer JD (1985). Comparative organization of chloroplast genomes. Annu Rev Genet.

[29] Khakhlova O, Bock R (2006). Elimination of deleterious mutations in plastid genomes by gene conversion. Plant J.

[30] Asano T, Tsudzuki T, Takahashi S, Shimada H, Kadowaki KI (2004). Complete nucleotide sequence of the sugarcane (*Saccharum officinarum*) chloroplast genome: a comparative analysis of four monocot chloroplast genomes. DNA Res.

[31] Gao L, Yi X, Yang YX, Su YJ, Wang T (2009). Complete chloroplast genome sequence of a tree fern *Alsophila spinulosa*: insights into evolutionary changes in fern chloroplast genomes. BMC Evol Biol.

[32] Maréchal A, Brisson N (2010). Recombination and the maintenance of plant organelle genome stability. New Phytol.

[33] Chen C, Zheng Y, Liu S, Zhong Y, Wu Y, Li J (2017). The complete chloroplast genome of *Cinnamomum camphora* and its comparison with related Lauraceae species. PeerJ..

[34] Wang ML, Barkley NA, Jenkins TM (2009). Microsatellite markers in plants and insects. Part I Appl Biotechnol G3.

[35] Kuang DY, Wu H, Wang YL, Gao LM, Zhang SZ, Lu L (2011). Complete chloroplast genome sequence of *Magnolia kwangsiensis* (Magnoliaceae): implication for DNA barcoding and population genetics. Genome..

[36] Gao R, Wang W, Huang Q, Fan R, Wang X, Feng P (2018). Complete chloroplast genome sequence of *Dryopteris fragrans* (L.) Schott and the repeat structures against the thermal environment. Sci Rep.

[37] Schneider H, Schuettpelz E, Pryer KM, Cranfill R, Magallón S, Lupia R (2004). Ferns diversified in the shadow of angiosperms. Nature..

[38] Milligan BG, Hampton JN, Palmer JD (1989). Dispersed repeats and structural reorganization in subclover chloroplast DNA. Mol Biol Evol.

[39] Odom OW, Baek KH, Dani RN, Herrin DL (2008). Chlamydomonas chloroplasts can use short dispersed repeats and multiple pathways to repair a double-strand break in the genome. Plant J.

[40] Andrews S. FastQC a quality control tool for high throughput sequence data. http://www.bioinformatics.babraham.ac.uk/projects/fastqc/.

[41] Bolger AM, Lohse M, Usadel B (2014). Trimmomatic: a flexible trimmer for Illumina sequence data. Bioinformatics..

[42] Zerbino DR, Birney E (2008). Velvet: algorithms for de novo short read assembly using de Bruijn graphs. Genome Res.

[43] Wyman SK, Jansen RK, Boore JL (2004). Automatic annotation of organellar genomes with DOGMA. Bioinformatics..

[44] Laslett D, Canback B (2004). ARAGORN, a program to detect tRNA genes and tmRNA genes in nucleotide sequences. Nucleic Acids Res.

[45] Schattner P, Brooks AN, Lowe TM (2005). The tRNAscan-SE, snoscan and snoGPS web servers for the detection of tRNAs and snoRNAs. Nucleic Acids Res.

[46] Lohse M, Drechsel O, Kahlau S, Bock R (2013). OrganellarGenomeDRAW—a suite of tools for generating physical maps of plastid and mitochondrial genomes and visualizing expression data sets. Nucleic Acids Res.

[47] Darling AC, Mau B, Blattner FR, Perna NT (2004). Mauve: multiple alignment of conserved genomic sequence with rearrangements. Genome Res.

[48] Frazer KA, Pachter L, Poliakov A, Rubin EM, Dubchak I (2004). VISTA: computational tools for comparative genomics. Nucleic Acids Res.

[49] Katoh K, Standley DM (2013). MAFFT multiple sequence alignment software version 7: improvements in performance and usability. Mol Biol Evol.

[50] Hall TA (1999). BioEdit: a user-friendly biological sequence alignment editor and analysis program for windows 95/98/NT. Nucleic Acids Symp Ser.

[51] Librado P, Rozas J (2009). DnaSP v5: a software for comprehensive analysis of DNA polymorphism data. Bioinformatics..

[52] Beier S, Thiel T, Münch T, Scholz U, Mascher M (2017). MISA-web: a web server for microsatellite prediction. Bioinformatics..

[53] Kurtz S, Choudhuri JV, Ohlebusch E, Schleiermacher C, Stoye J, Giegerich R (2001). REPuter: the manifold applications of repeat analysis on a genomic scale. Nucleic Acids Res.

