# Supplementary material for: Comparative genomic analysis of Polypodiaceae chloroplasts reveals fine structural features and dynamic insertion sequences
Source: BMC Plant Biol. 2021 Jan 7;21:31. doi: 10.1186/s12870-020-02800-x (PMC7792340; doi:10.1186/s12870-020-02800-x)
Supplement: Supplementary file 1 — Additional file 1: Table S1. Primers used for plastomes gap closing. Table S2. Distribution of SSRs in different plastome regions of Polypodiaceae. Table S3. Information on dispersed repeats among Polypodiaceae plastomes. [file 12870_2020_2800_MOESM1_ESM.docx]

Table S1. Primers used for plastomes gap closing.

| Primer | Forward sequence | Reverse sequence |
| --- | --- | --- |
| JNXJ1 | 5'GTAATCGTCTTCAGTAGGG3' | 5'AATGCTCGTTACCCCAC3' |
| JNXJ2 | 5'CCAGTGATTCGGAGGTTG3' | 5'GGGGAGCGAATGGGATTA3' |
| JNXJ3 | 5'ACGAAGTAATCCCCACAG3' | 5'AGCGTGGTAGAATAGCCG3' |
| DJ1 | 5'GTACCCCTAGTTGGTTTG3' | 5'GCACCGCAGACAAGGATA3' |
| DJ2 | 5'TTTACCCCAGTCGTCATTT3' | 5'AAGAAGCAAGGGGTAAGG3' |
| GLLJ1 | 5'AGAGGGCAAGATACGAGG3' | 5'ATAGTCCTTCCCCTCCTG3' |

| GLLJ2 | 5'ATCGTTTTGGTTCCTCCTC3' | 5'ATAGGCGGATAGCGGGA3' |
| --- | --- | --- |
| GLLJ3 | 5'GTATCTGCCTATCTCCTTC3' | 5'AGCACTTGTATTACCCGAA3' |
| GLLJ4 | 5'ATCGGAGCGGTGTAGTGA3' | 5'TAAGACACCGAACCATCC3' |
| GLLJ5 | 5'CAGTCTCGTACACACTCAA3' | 5'ACTAACTATGAAAACTCGCC3' |
| GLLJ6 | 5'CAAGAATCCCGTAAGAGTG3' | 5'CAGTCTCGTACACACTCAA3' |

JNXJ, *N. fortune*; DJ, *N. ovatus*; GLLJ, *P. cuspidatus*.

Table S2. Distribution of SSRs in different plastome regions of Polypodiaceae.

| Species | CDS | intergenic | intronic | LSC | IR | SSC |
| --- | --- | --- | --- | --- | --- | --- |
| *N. ovatus* | 4.3% | 71.7% | 23.9% | 67.4% | 21.7% | 10.9% |
| *N. fortunei* | 8.7% | 76.1% | 15.2% | 63.0% | 21.7% | 15.2% |
| *P. cuspidatus* | 8.9% | 71.1% | 20.0% | 57.8% | 31.1% | 11.1% |
| *P. niponica* | 9.3% | 74.4% | 16.3% | 67.4% | 23.3% | 9.3% |
| *L. clathratus* | 8.9% | 66.7% | 24.4% | 68.9% | 13.3% | 17.8% |
| *L. hemionitideus* | 2.2% | 68.9% | 28.9% | 57.8% | 22.2% | 20.0% |
| *L. microphyllum* | 10.5% | 71.1% | 18.4% | 71.1% | 10.5% | 18.4% |
| *L. hederaceum* | 7.5% | 67.5% | 25.0% | 65.0% | 25.0% | 10.0% |
| *P. bifurcatum* | 7.1% | 83.3% | 9.5% | 64.3% | 14.3% | 21.4% |
| *P. bonii* | 4.0% | 74.0% | 22.0% | 54.0% | 24.0% | 22.0% |
| *D. roosii* | 11.8% | 74.5% | 13.7% | 54.9% | 27.5% | 17.6% |
| *S. yakushimensis* | 14.0% | 76.0% | 10.0% | 48.0% | 36.0% | 16.0% |

Table S3. Information on dispersed repeats among Polypodiaceae plastomes.

| ***N. fortunei*** | | | | | |
| --- | --- | --- | --- | --- | --- |
| No. | Size | Type | Start 1 | Start 2 | Location |
| 1 | 30 | F | 52609 | 52624 | *trn*R-UCG-*acc*D |
| 2 | 30 | F | 27337 | 27363 | *psb*M-*pet*N |
| 3 | 31 | F | 116588 | 116618 | *ndh*A intron |
| 4 | 44 | F | 116588 | 116603 | *ndh*A intron |
| 5 | 30 | P | 29672 | 29707 | *trn*S-UGA-*psb*C |
| 6 | 32 | P | 47953 | 47953 | *trn*V-UAC-*trn*M-CAU |
| 7 | 34 | P | 68655 | 68655 | *psb*T-*psb*N |
| 8 | 35 | P | 34788 | 34830 | *rps*14-*psa*B |
| 9 | 36 | P | 27020 | 27020 | *psb*M-*pet*N |
| 10 | 40 | P | 109150 | 109150 | *rpl*32-*trn*P-GGG |
| 11 | 46 | P | 18555 | 18555 | *rps*12-*rpoC*1 |
| 12 | 52 | P | 75036 | 75036 | *rps*8-*rpl*14 |
| 13 | 67 | P | 1786 | 1786 | *mat*K-*rps*16 |
| ***N. ovatus*** | | | | | |
| No. | Size | Type | Start 1 | Start 2 | Location |
| 1 | 30 | F | 108601 | 108624 | *rpl*21-*rpl*32 |
| 2 | 30 | F | 36889 | 39113 | *psa*B/*psa*A |
| 3 | 31 | F | 83869 | 149156 | *trn*T-UGU intron |
| 4 | 32 | F | 126433 | 126443 | *chl*N-*chl*L |
| 5 | 32 | F | 99652 | 133375 | *ycf*2 |
| 6 | 34 | F | 41422 | 138377 | *rps*12-*rrn*16 |
| 7 | 38 | F | 83879 | 83917 | *trn*T-UGU intron |
| 8 | 38 | F | 149098 | 149136 | *trn*T-UGU intron |
| 9 | 57 | F | 83879 | 83898 | *trn*T-UGU intron |
| 10 | 57 | F | 149098 | 149117 | *trn*T-UGU intron |
| 11 | 32 | P | 1900 | 1937 | *mat*K-*rps*16 |
| 12 | 34 | P | 41422 | 94642 | *ycf*3 intron |
| 13 | 36 | P | 27661 | 27661 | *psb*M-*pet*N |
| 14 | 38 | P | 30277 | 30320 | *trn*S-UGA-*psb*C |
| 15 | 38 | P | 83879 | 149098 | *trn*T-UGU intron |
| 16 | 38 | P | 83917 | 149136 | *trn*T-UGU intron |
| 17 | 57 | P | 83879 | 149098 | *trn*T-UGU intron |
| 18 | 57 | P | 83898 | 149117 | *trn*T-UGU intron |
| 19 | 30 | R | 113136 | 113136 | *ndh*D-*psa*C |
| ***P. cuspidatum*** | | | | | |
| No. | Size | Type | Start 1 | Start 2 | Location |
| 1 | 30 | F | 113804 | 113819 | *psa*C-*ndh*E |
| 2 | 30 | F | 36938 | 39162 | *psa*B/*psa*A |
| 3 | 31 | F | 1733 | 1778 | *mat*K-*rps*16 |
| 4 | 31 | F | 2178 | 2217 | *mat*K-*rps*16 |
| 5 | 32 | F | 99726 | 133585 | *ycf*2/*ycf*2 |
| 6 | 34 | F | 41471 | 138605 | *ycf*3 intron/*rps*12 intron |
| 7 | 44 | F | 2178 | 2204 | *mat*K-*rps*16 |
| 8 | 46 | F | 1718 | 1763 | *mat*K-*rps*16 |
| 9 | 58 | F | 2177 | 2190 | *mat*K-*rps*16 |
| 10 | 30 | P | 1943 | 1978 | *mat*K-*rps*16 |
| 11 | 30 | P | 60649 | 60649 | *psb*E-*pet*L |
| 12 | 32 | P | 6700 | 30176 | *psb*I-*trn*S-GCU/*trn*S-UGA |
| 13 | 34 | P | 69229 | 69229 | *psb*T-*psb*N |
| 14 | 34 | P | 41471 | 94697 | *ycf*3 intron/*rrn*16-*rps*12 |
| 15 | 35 | P | 30316 | 30359 | *trn*S-UGA-*psb*C |
| 16 | 36 | P | 19362 | 19362 | *rpoC*2-*rpoC*1 |
| 17 | 36 | P | 27806 | 27806 | *psb*M-*pet*N |
| 18 | 52 | P | 75589 | 75589 | *rps*8-*rpl*14 |
| 19 | 307 | P | 105517 | 127512 | *trn*N-*ndh*F/*chl*L-*trn*N |
| ***P. niponica*** | | | | | |
| No. | size | type | Start 1 | Start 2 | Location |
| 1 | 31 | F | 47465 | 47474 | *ndh*C-*trn*V-UAC |
| 2 | 31 | F | 108504 | 108506 | *ndh*F-*rpl*21 |
| 3 | 36 | F | 25926 | 25944 | *rpo*B-*trn*D-GUC |
| 4 | 60 | F | 53388 | 53403 | *trn*R-UCG-*acc*D |
| 5 | 30 | P | 30400 | 30435 | *trn*S-UGA-*psb*C |
| 6 | 30 | P | 108507 | 108507 | *ndh*F-*rpl*21 |
| 7 | 30 | P | 108506 | 108506 | *ndh*F-*rpl*21 |
| 8 | 34 | P | 69471 | 69471 | *psb*D-*psb*N |
| 9 | 36 | P | 35527 | 35568 | *rps*14-*psa*B |
| 10 | 36 | P | 27736 | 27736 | *psb*M-*pet*N |
| 11 | 46 | P | 19238 | 19238 | *rpoC*2-*rpoC*1 |
| 12 | 52 | P | 75866 | 75866 | *rps*8-*rpl*14 |
| 13 | 69 | P | 1915 | 1915 | *mat*K-*rps*16 |
| ***L. clathratus*** | | | | | |
| No. | Size | Type | Start 1 | Start 2 | Location |
| 1 | 43 | F | 144740 | 144758 | *rps*12*-rrn*16 |
| 2 | 43 | F | 144749 | 144758 | *rps*12*-rrn*16 |
| 3 | 50 | F | 93290 | 93326 | *rps*12*-rrn*16 |
| 4 | 50 | F | 93290 | 143141 | *rrn*16-*rps*12/*rps*12*-rrn*16 |
| 5 | 50 | F | 94900 | 144751 | *rrn*16-*rps*12/*rps*12*-rrn*16 |
| 6 | 50 | F | 144715 | 144751 | *rps*12*-rrn*16 |
| 7 | 51 | F | 93298 | 143140 | *rrn*16-*rps*12/*rps*12*-rrn*16 |
| 8 | 51 | F | 94908 | 144750 | *rrn*16-*rps*12/*rps*12*-rrn*16 |
| 9 | 52 | F | 93271 | 143122 | *rrn*16-*rps*12/*rps*12*-rrn*16 |
| 10 | 52 | F | 94917 | 144768 | *rrn*16-*rps*12/*rps*12*-rrn*16 |
| 11 | 59 | F | 144715 | 144742 | *rps*12*-rrn*16 |
| 12 | 68 | F | 93290 | 93299 | *rrn*16-*rps*12 |
| 13 | 68 | F | 144724 | 144733 | *rps*12*-rrn*16 |
| 14 | 44 | P | 19255 | 19255 | *rpoC*2-*rpoC*1 |
| 15 | 50 | P | 93290 | 94900 | *rrn*16-*rps*12 |
| 16 | 50 | P | 93290 | 144715 | *rrn*16-*rps*12/*rps*12*-rrn*16 |
| 17 | 50 | P | 93326 | 144751 | *rrn*16-*rps*12/*rps*12*-rrn*16 |
| 18 | 50 | P | 143141 | 144751 | *rps*12*-rrn*16 |
| 19 | 51 | P | 93298 | 94900 | *rrn*16-*rps*12 |
| 20 | 51 | P | 143140 | 144742 | *rps*12-*rrn*16 |
| 21 | 52 | P | 75459 | 75459 | *rps*8-*rpl*14 |
| 22 | 52 | P | 93271 | 94917 | *rrn*16-*rps*12 |
| 23 | 52 | P | 143122 | 144768 | *rps*12-*rrn*16 |
| 24 | 59 | P | 93290 | 144715 | *rps*12-*rrn*16 |
| 25 | 59 | P | 93308 | 144742 | *rrn*16-*rps*12/*rps*12-*rrn*16 |

| 26 | 59 | P | 93317 | 144742 | | *rrn*16-*rps*12/*rps*12-*rrn*16 |
| --- | --- | --- | --- | --- | --- | --- |
| 27 | 68 | P | 93290 | 144724 | | *rrn*16-*rps*12/*rps*12-*rrn*16 |
| 28 | 68 | P | 93299 | 144733 | | *rrn*16-*rps*12/*rps*12-*rrn*16 |
| 29 | 69 | P | 1948 | 1948 | | *mat*K-*rps*16 |
| 30 | 40 | R | 25543 | 25543 | | *rpo*B-*trn*D-GUC |
| ***L. hemionitideus*** | | | | | | |
| No. | Size | Type | Start 1 | Start 2 | | Location |
| 1 | 34 | F | 121879 | 121888 | | *rps*15-*ycf*1 |
| 2 | 30 | P | 19369 | 19369 | | *rpoC*2-*rpoC*1 |
| 3 | 30 | P | 60800 | 60800 | | *psb*E-*pet*L |
| 4 | 32 | P | 30444 | 30495 | | *trn*S-UGA-*psb*C |
| 5 | 34 | P | 69359 | 69359 | | *psb*T-*psb*N |
| 6 | 36 | P | 27806 | 27806 | | *psb*M-*pet*N |
| 7 | 48 | P | 108358 | 108358 | | *ndh*F-*rpl*21 |
| 8 | 52 | P | 75742 | 75742 | | *rps*8-*rpl*14 |
| 9 | 34 | R | 51201 | 51201 | | *atp*B-*rbc*L |
| ***L. microphyllum*** | | | | | | |
| No. | size | Type | start | start | | location |
| 1 | 30 | F | 12810 | 12840 | | *atp*H-*atp*I |
| 2 | 37 | F | 97068 | 97086 | | *rps*7-*psb*A |
| 3 | 32 | F | 98560 | 98585 | | *rps*7-*psb*A |
| 4 | 52 | F | 98748 | 98869 | | *rps*7-*psb*A |
| 5 | 54 | F | 98748 | 98896 | | *rps*7-*psb*A |
| 6 | 34 | F | 98768 | 98916 | | *rps*7-*psb*A |
| 7 | 30 | F | 98775 | 98869 | | *rps*7-*psb*A |
| 8 | 32 | F | 98787 | 98935 | | *rps*7-*psb*A |
| 9 | 33 | F | 98816 | 99073 | | *rps*7-*psb*A |
| 10 | 52 | F | 98869 | 98896 | | *rps*7-*psb*A |
| 11 | 32 | F | 98889 | 98916 | | *rps*7-*psb*A |
| 12 | 34 | F | 99059 | 99068 | | *rps*7-*psb*A |
| 13 | 34 | F | 99059 | 142163 | | *rps*7-*psb*A |
| 14 | 32 | F | 99064 | 142150 | | *rps*7-*psb*A |
| 15 | 35 | F | 99067 | 142162 | | *rps*7-*psb*A |
| 16 | 31 | F | 99071 | 142148 | | *rps*7-*psb*A |
| 17 | 32 | F | 102657 | 136590 | | *ycf*2 |
| 18 | 58 | F | 119959 | 119974 | | *ndh*A intron |
| 19 | 45 | F | 119959 | 119989 | | *ndh*A intron |
| 20 | 30 | F | 119959 | 120004 | | *ndh*A intron |
| 21 | 33 | F | 140167 | 140424 | | *psb*A-*rps*7 |
| 22 | 34 | F | 140171 | 140180 | | *psb*A-*rps*7 |
| 23 | 32 | F | 140306 | 140454 | | *psb*A-*rps*7 |
| 24 | 54 | F | 140323 | 140471 | | *psb*A-*rps*7 |
| 25 | 52 | F | 140325 | 140352 | | *psb*A-*rps*7 |
| 26 | 37 | F | 140340 | 140367 | | *psb*A-*rps*7 |
| 27 | 37 | F | 140340 | 140488 | | *psb*A-*rps*7 |
| 28 | 52 | F | 140352 | 140473 | | *psb*A-*rps*7 |
| 29 | 38 | F | 140366 | 140487 | | *psb*A-*rps*7 |
| 30 | 30 | F | 140374 | 140468 | | *psb*A-*rps*7 |
| 31 | 30 | F | 140470 | 140497 | | *psb*A-*rps*7 |
| 32 | 32 | F | 140656 | 140681 | | *psb*A-*rps*7 |
| 33 | 37 | F | 142150 | 142168 | | *psb*A-*rps*7 |
| 34 | 59 | P | 1948 | 1948 | | *mat*K-*rps*16 |
| 35 | 36 | P | 27689 | 27689 | | *psb*M-*pet*N |
| 36 | 38 | P | 30230 | 30273 | | *trn*S-*psb*C |
| 37 | 75 | P | 35383 | 35383 | | *rps*14-*psa*B |
| 38 | 34 | P | 69233 | 69233 | | *psb*T-*psb*N |
| 39 | 54 | P | 75587 | 75587 | | *rps*8-*rpl*14 |
| 40 | 37 | P | 97068 | 142150 | | *rps*7-*psb*A |
| 41 | 32 | P | 98560 | 140656 | | *rps*7-*psb*A |
| 42 | 52 | P | 98748 | 140352 | | *rps*7-*psb*A |
| 43 | 54 | P | 98748 | 140323 | | *rps*7-*psb*A |
| 44 | 30 | P | 98748 | 140468 | | *rps*7-*psb*A |
| 45 | 34 | P | 98768 | 140323 | | *rps*7-*psb*A |
| 46 | 30 | P | 98775 | 140374 | | *rps*7-*psb*A |
| 47 | 30 | P | 98775 | 140495 | | *rps*7-*psb*A |
| 48 | 32 | P | 98787 | 140306 | | *rps*7-*psb*A |
| 49 | 33 | P | 98816 | 140167 | | *rps*7-*psb*A |
| 50 | 52 | P | 98869 | 140473 | | *rps*7-*psb*A |
| 51 | 52 | P | 98869 | 140325 | | *rps*7-*psb*A |
| 52 | 30 | P | 98869 | 140468 | | *rps*7-*psb*A |
| 53 | 32 | P | 98889 | 140325 | | *rps*7-*psb*A |
| 54 | 54 | P | 98896 | 140471 | | *rps*7-*psb*A |
| 55 | 52 | P | 98896 | 140352 | | *rps*7-*psb*A |
| 56 | 34 | P | 98916 | 140471 | | *rps*7-*psb*A |
| 57 | 32 | P | 98916 | 140352 | | *rps*7-*psb*A |
| 58 | 32 | P | 98935 | 140454 | | *rps*7-*psb*A |
| 59 | 34 | P | 99059 | 140171 | | *rps*7-*psb*A |
| 60 | 34 | P | 99068 | 140180 | | *rps*7-*psb*A |
| 61 | 33 | P | 99073 | 140424 | | *rps*7-*psb*A |
| 62 | 35 | P | 140171 | 142162 | | *psb*A-*rps*7 |
| 63 | 34 | P | 140180 | 142163 | | *psb*A-*rps*7 |
| ***L. hederaceum*** | | | | | | |
| No. | Size | Type | Start 1 | Start 2 | | Location |
| 1 | 34 | F | 19279 | 48627 | | *rpoC*2-*rpoC*1 |
| 2 | 31 | F | 19280 | 19282 | | *rpoC*2-*rpoC*1 |
| 3 | 31 | F | 19282 | 48628 | | *rpoC*2-*rpoC*1 |
| 4 | 30 | F | 36978 | 39202 | | *psa*B |
| 5 | 34 | F | 48631 | 48637 | | *trn*V-UAC intron |
| 6 | 32 | F | 48633 | 48635 | | *trn*V-UAC intron |
| 7 | 30 | F | 48635 | 48645 | | *trn*V-UAC intron |
| 8 | 31 | F | 48637 | 48645 | | *trn*V-UAC intron |
| 9 | 32 | F | 99901 | 133805 | | *ycf*2 |
| 10 | 67 | P | 1936 | 1936 | | *mat*K-*rps*16 |
| 11 | 31 | P | 19280 | 19283 | | *rpoC*2-*rpoC*1 |
| 12 | 31 | P | 19282 | 19285 | | *rpoC*2-*rpoC*1 |
| 13 | 36 | P | 27746 | 27746 | | *psb*M-*pet*N |
| 14 | 30 | P | 30350 | 30385 | | *trn*S-*psb*C |
| 15 | 40 | P | 48631 | 48631 | | *trn*V-UAC intron |
| 16 | 32 | P | 48639 | 48639 | | *trn*V-UAC intron |
| 17 | 52 | P | 75725 | 75725 | | *rps*8-*rpl*14 |
| 18 | 31 | R | 19281 | 19281 | | *rpoC*2-*rpoC*1 |
| 19 | 31 | R | 19281 | 48635 | | *rpoC*2-*rpoC*1 |
| 20 | 30 | R | 35381 | 35381 | | *rps*14 |
| 21 | 30 | R | 48636 | 48637 | | *trn*V-UAC intron |
| 22 | 30 | R | 48637 | 48638 | | *trn*V-UAC intron |
| 23 | 30 | R | 48637 | 48640 | | *trn*V-UAC intron |
| 24 | 33 | R | 48638 | 48638 | | *trn*V-UAC intron |
| 25 | 33 | R | 48638 | 48642 | | *trn*V-UAC intron |
| 26 | 31 | R | 48640 | 48640 | | *trn*V-UAC intron |
| ***P. bifurcatum*** | | | | | | |
| No. | Size | Type | Start 1 | Start 2 | | Location |
| 1 | 31 | F | 35761 | 37985 | | *psa*B |
| 2 | 31 | F | 35761 | 37985 | | *psa*B |
| 3 | 35 | F | 36519 | 38725 | | *psa*B |
| 4 | 34 | F | 40371 | 139847 | | *ycf*3 intron |
| 5 | 30 | F | 81790 | 154167 | | *trn*T-UGU intron |
| 6 | 49 | F | 91815 | 142636 | | *rrn1*6-*rps*12 |
| 7 | 49 | F | 91815 | 142645 | | *rrn1*6-*rps*12 |
| 8 | 49 | F | 91815 | 142654 | | *rrn1*6-*rps*12 |
| 9 | 49 | F | 91815 | 142663 | | *rrn1*6-*rps*12 |
| 10 | 49 | F | 91815 | 142672 | | *rrn1*6-*rps*12 |
| 11 | 47 | F | 91815 | 142681 | | *rrn1*6-*rps*12 |
| 12 | 40 | F | 91815 | 91824 | | *rrn1*6-*rps*12 |
| 13 | 31 | F | 91815 | 142753 | | *rrn1*6-*rps*12 |
| 14 | 36 | F | 91815 | 142699 | | *rrn1*6-*rps*12 |
| 15 | 48 | F | 93203 | 93320 | | *rrn1*6-*rps*12 |
| 16 | 35 | F | 93203 | 93266 | | *rrn1*6-*rps*12 |
| 17 | 35 | F | 93203 | 93275 | | *rrn1*6-*rps*12 |
| 18 | 35 | F | 93203 | 93284 | | *rrn1*6-*rps*12 |
| 19 | 35 | F | 93203 | 93293 | | *rrn1*6-*rps*12 |
| 20 | 35 | F | 93203 | 93302 | | *rrn1*6-*rps*12 |
| 21 | 35 | F | 93203 | 93311 | | *rrn1*6-*rps*12 |
| 22 | 35 | F | 93203 | 144123 | | *rrn1*6-*rps*12 |
| 23 | 35 | F | 93203 | 144132 | | *rrn1*6-*rps*12 |
| 24 | 31 | F | 93203 | 144141 | | *rrn1*6-*rps*12 |
| 25 | 35 | F | 93203 | 93329 | | *rrn1*6-*rps*12 |
| 26 | 31 | F | 93203 | 93212 | | *rrn1*6-*rps*12 |
| 27 | 33 | F | 93205 | 93259 | | *rrn1*6-*rps*12 |
| 28 | 31 | F | 93207 | 93252 | | *rrn1*6-*rps*12 |
| 29 | 31 | F | 93212 | 144123 | | *rrn1*6-*rps*12 |
| 30 | 36 | F | 93252 | 144136 | | *rrn1*6-*rps*12 |
| 31 | 87 | F | 93259 | 93268 | | *rrn1*6-*rps*12 |
| 32 | 78 | F | 93259 | 93277 | | *rrn1*6-*rps*12 |
| 33 | 69 | F | 93259 | 93286 | | *rrn1*6-*rps*12 |
| 34 | 60 | F | 93259 | 93295 | | *rrn1*6-*rps*12 |
| 35 | 51 | F | 93259 | 93304 | | *rrn1*6-*rps*12 |
| 36 | 47 | F | 93259 | 144125 | | *rrn1*6-*rps*12 |
| 37 | 42 | F | 93259 | 93313 | | *rrn1*6-*rps*12 |
| 38 | 38 | F | 93259 | 144134 | | *rrn1*6-*rps*12 |
| 39 | 33 | F | 93259 | 93322 | | *rrn1*6-*rps*12 |
| 40 | 35 | F | 93259 | 93331 | | *rrn1*6-*rps*12 |
| 41 | 49 | F | 93266 | 144123 | | *rrn1*6-*rps*12 |
| 42 | 49 | F | 93275 | 144123 | | *rrn1*6-*rps*12 |
| 43 | 49 | F | 93284 | 144123 | | *rrn1*6-*rps*12 |
| 44 | 49 | F | 93293 | 144123 | | *rrn1*6-*rps*12 |
| 45 | 49 | F | 93302 | 144123 | | *rrn1*6-*rps*12 |
| 46 | 44 | F | 93311 | 144123 | | *rrn1*6-*rps*12 |
| 47 | 35 | F | 93320 | 144123 | | *rrn1*6-*rps*12 |
| 48 | 37 | F | 93329 | 144123 | | *rrn1*6-*rps*12 |
| 49 | 40 | F | 97568 | 138379 | | *rps*7-*psb*A |
| 50 | 32 | F | 101123 | 134838 | | *ycf*2 |
| 51 | 30 | F | 102284 | 102299 | | *ycf*2 |
| 52 | 30 | F | 133660 | 133675 | | *ycf*2 |
| 53 | 48 | F | 142619 | 142736 | | *rps*12-*rrn*16 |
| 54 | 35 | F | 142621 | 142693 | | *rps*12-*rrn*16 |
| 55 | 35 | F | 142623 | 142749 | | *rps*12-*rrn*16 |
| 56 | 87 | F | 142632 | 142641 | | *rps*12-*rrn*16 |
| 57 | 78 | F | 142632 | 142650 | | *rps*12-*rrn*16 |
| 58 | 69 | F | 142632 | 142659 | | *rps*12-*rrn*16 |
| 59 | 60 | F | 142632 | 142668 | | *rps*12-*rrn*16 |
| 60 | 51 | F | 142632 | 142677 | | *rps*12-*rrn*16 |
| 61 | 42 | F | 142632 | 142686 | | *rps*12-*rrn*16 |
| 62 | 33 | F | 142632 | 142695 | | *rps*12-*rrn*16 |
| 63 | 35 | F | 142641 | 142749 | | *rps*12-*rrn*16 |
| 64 | 35 | F | 142650 | 142749 | | *rps*12-*rrn*16 |
| 65 | 35 | F | 142659 | 142749 | | *rps*12-*rrn*16 |
| 66 | 35 | F | 142668 | 142749 | | *rps*12-*rrn*16 |
| 67 | 35 | F | 142677 | 142749 | | *rrn1*6-*rps*12 |
| 68 | 35 | F | 142686 | 142749 | | *rrn1*6-*rps*12 |
| 69 | 33 | F | 142695 | 142749 | | *rrn1*6-*rps*12 |
| 70 | 31 | F | 142704 | 142749 | | *rrn1*6-*rps*12 |
| 71 | 31 | F | 142749 | 142758 | | *rrn1*6-*rps*12 |
| 72 | 40 | F | 144123 | 144132 | | *rrn1*6-*rps*12 |
| 73 | 31 | F | 144123 | 144141 | | *rrn1*6-*rps*12 |
| 74 | 30 | P | 5897 | 29011 | | *psb*I-*trn*S/*trn*S-GUA |
| 75 | 42 | P | 18245 | 18245 | | *rpoC*2-*rpoC*1 |
| 76 | 35 | P | 29193 | 29239 | | *trn*S-*psb*C |
| 77 | 34 | P | 40371 | 96106 | | *ycf3* intron |
| 78 | 34 | P | 62294 | 62294 | | *rpl2*0-*rps*12 |
| 79 | 30 | P | 81790 | 81790 | | *trn*T-UGU intron |
| 80 | 49 | P | 91815 | 93266 | | *rrn1*6-*rps*12 |
| 81 | 49 | P | 91815 | 93275 | | *rrn1*6-*rps*12 |
| 82 | 49 | P | 91815 | 93284 | | *rrn1*6-*rps*12 |
| 83 | 49 | P | 91815 | 93293 | | *rrn1*6-*rps*12 |
| 84 | 49 | P | 91815 | 93302 | | *rrn1*6-*rps*12 |
| 85 | 47 | P | 91815 | 93259 | | *rrn1*6-*rps*12 |
| 86 | 40 | P | 91815 | 144123 | | *rrn1*6-*rps*12 |
| 87 | 48 | P | 93203 | 142619 | | *rrn1*6-*rps*12 |
| 88 | 35 | P | 93203 | 142641 | | *rrn1*6-*rps*12 |
| 89 | 35 | P | 93203 | 142650 | | *rrn1*6-*rps*12 |
| 90 | 35 | P | 93203 | 142659 | | *rrn1*6-*rps*12 |
| 91 | 35 | P | 93203 | 142668 | | *rrn1*6-*rps*12 |
| 92 | 35 | P | 93203 | 142677 | | *rrn1*6-*rps*12 |
| 93 | 35 | P | 93203 | 142686 | | *rrn1*6-*rps*12 |
| 94 | 35 | P | 93203 | 142623 | | *rrn1*6-*rps*12 |
| 95 | 31 | P | 93203 | 142744 | | *rrn1*6-*rps*12 |
| 96 | 33 | P | 93205 | 142695 | | *rrn1*6-*rps*12 |
| 97 | 31 | P | 93207 | 142704 | | *rrn1*6-*rps*12 |
| 98 | 31 | P | 93212 | 142753 | | *rrn1*6-*rps*12 |
| 99 | 31 | P | 93252 | 142749 | | *rrn1*6-*rps*12 |
| 100 | 87 | P | 93259 | 142632 | | *rrn1*6-*rps*12 |
| 101 | 78 | P | 93259 | 142632 | | *rrn1*6-*rps*12 |
| 102 | 69 | P | 93259 | 142632 | | *rrn1*6-*rps*12 |
| 103 | 60 | P | 93259 | 142632 | | *rrn1*6-*rps*12 |
| 104 | 51 | P | 93259 | 142632 | | *rrn1*6-*rps*12 |
| 105 | 42 | P | 93259 | 142632 | | *rrn1*6-*rps*12 |
| 106 | 33 | P | 93259 | 142632 | | *rrn1*6-*rps*12 |
| 107 | 33 | P | 93259 | 142749 | | *rrn1*6-*rps*12 |
| 108 | 35 | P | 93259 | 142621 | | *rrn1*6-*rps*12 |
| 109 | 35 | P | 93266 | 142749 | | *rrn1*6-*rps*12 |
| 110 | 87 | P | 93268 | 142641 | | *rrn1*6-*rps*12 |
| 111 | 35 | P | 93275 | 142749 | | *rrn1*6-*rps*12 |
| 112 | 78 | P | 93277 | 142650 | | *rrn1*6-*rps*12 |
| 113 | 35 | P | 93284 | 142749 | | *rrn1*6-*rps*12 |
| 114 | 69 | P | 93286 | 142659 | | *rrn1*6-*rps*12 |
| 115 | 35 | P | 93293 | 142749 | | *rrn1*6-*rps*12 |
| 116 | 60 | P | 93295 | 142668 | | *rrn1*6-*rps*12 |
| 117 | 35 | P | 93302 | 142749 | | *rrn1*6-*rps*12 |
| 118 | 51 | P | 93304 | 142677 | | *rrn1*6-*rps*12 |
| 119 | 35 | P | 93311 | 142749 | | *rrn1*6-*rps*12 |
| 120 | 42 | P | 93313 | 142686 | | *rrn1*6-*rps*12 |
| 121 | 48 | P | 93320 | 142736 | | *rrn1*6-*rps*12 |
| 122 | 33 | P | 93322 | 142695 | | *rrn1*6-*rps*12 |
| 123 | 35 | P | 93329 | 142749 | | *rrn1*6-*rps*12 |
| 124 | 35 | P | 93331 | 142693 | | *rrn1*6-*rps*12 |
| 125 | 40 | P | 97568 | 97568 | | *rps*7-*psb*A |
| 126 | 30 | P | 102284 | 133658 | | *ycf*2 |
| 127 | 30 | P | 102299 | 133673 | | *ycf*2 |
| 128 | 30 | P | 109600 | 109600 | | *ndh*F-*rpl*21 |
| 129 | 52 | P | 110964 | 110964 | | *rpl*32-*trn*P |
| 130 | 40 | P | 138379 | 138379 | | *psb*A-*rps*7 |
| 131 | 37 | P | 142621 | 144123 | | *rps*12-*rrn*16 |
| 132 | 44 | P | 142632 | 144123 | | *rps*12-*rrn*16 |
| 133 | 35 | P | 142632 | 144123 | | *rps*12-*rrn*16 |
| 134 | 49 | P | 142636 | 144123 | | *rps*12-*rrn*16 |
| 135 | 49 | P | 142645 | 144123 | | *rps*12-*rrn*16 |
| 136 | 49 | P | 142654 | 144123 | | *rps*12-*rrn*16 |
| 137 | 49 | P | 142663 | 144123 | | *rps*12-*rrn*16 |
| 138 | 49 | P | 142672 | 144123 | | *rps*12-*rrn*16 |
| 139 | 47 | P | 142681 | 144125 | | *rrn1*6-*rps*12 |
| 140 | 38 | P | 142690 | 144134 | | *rrn1*6-*rps*12 |
| 141 | 36 | P | 142699 | 144136 | | *rrn1*6-*rps*12 |
| 142 | 35 | P | 142749 | 144123 | | *rrn1*6-*rps*12 |
| 143 | 35 | P | 142749 | 144132 | | *rrn1*6-*rps*12 |
| 144 | 31 | P | 142749 | 144118 | | *rrn1*6-*rps*12 |
| 145 | 31 | P | 142753 | 144141 | | *rrn1*6-*rps*12 |
| 146 | 30 | P | 154167 | 154167 | | *trn*T-UGU intron |
| ***P. bonii*** | | | | | | |
| No. | Size | Type | Start 1 | Start 2 | | Location |
| 1 | 30 | F | 85234 | 155389 | | *trn*T-UGU intron |
| 2 | 30 | F | 48020 | 111821 | | *trn*V-*trn*M /*ndh*F-*rpl*21 |
| 3 | 30 | F | 48024 | 111819 | | *trn*V-*trn*M/*ndh*F-*rpl*21 |
| 4 | 30 | F | 48026 | 111817 | | *trn*V-*trn*M/*ndh*F-*rpl*21 |
| 5 | 31 | F | 58224 | 58233 | | *pet*A-*psb*J |
| 6 | 31 | F | 35541 | 37777 | | *psa*B/*psa*A |
| 7 | 31 | F | 59684 | 59693 | | *pet*A-*psb*J |
| 8 | 32 | F | 103354 | 137273 | | *ycf*2 |
| 9 | 32 | F | 120072 | 120095 | | *ndh*A intron |
| 10 | 36 | F | 40928 | 142284 | | *ycf*3 intron/*rps*12-*rrn*16 |
| 11 | 40 | F | 99797 | 140816 | | *rps*7-*psb*A/*psb*A-*rps*7 |
| 12 | 30 | P | 85234 | 85234 | | *trn*T-UGU intron |
| 13 | 30 | P | 155389 | 155389 | | *trn*T-UGU intron |
| 14 | 30 | P | 48020 | 111815 | | *trn*V-*trn*M/*ndh*F-*rpl*21 |
| 15 | 30 | P | 48020 | 111819 | | *trn*V-*trn*M/*ndh*F-*rpl*21 |
| 16 | 30 | P | 48024 | 111821 | | *trn*V-*trn*M/*ndh*F-*rpl*21 |
| 17 | 30 | P | 58238 | 59694 | | *pet*A-*psb*J |
| 18 | 30 | P | 118759 | 118821 | | *ndh*G-*ndh*I |
| 19 | 31 | P | 58224 | 59689 | | *pet*A-*psb*J |
| 20 | 34 | P | 29815 | 29815 | | *trn*S-UGA-*psb*C |
| 21 | 34 | P | 70497 | 70497 | | *psb*T-*psb*N |
| 22 | 36 | P | 58232 | 59685 | | *pet*A-*psb*J |
| 23 | 36 | P | 40928 | 98333 | | *ycf*3 intron/*rrn*16-*rps*12 |
| 24 | 38 | P | 48018 | 48018 | | *trn*V-*trn*M |
| 25 | 40 | P | 99797 | 99797 | | *rps*7-*psb*A |
| 26 | 40 | P | 140816 | 140816 | | *psb*A-*rps*7 |
| 27 | 54 | P | 76835 | 76835 | | *rps*8-*rpl*14 |
| ***D. roosii*** | | | | | | |
| No. | Size | Type | Start 1 | Start 2 | | Location |
| 1 | 30 | F | 55744 | 55838 | | *rbc*L-*trn*R-UCG |
| 2 | 30 | F | 49529 | 49531 | | *trn*V-UAC-*trn*M-CAU |
| 3 | 30 | F | 37916 | 40140 | | *psa*B/psaA |
| 4 | 31 | F | 103360 | 136960 | | *ycf*2 |
| 5 | 31 | F | 20249 | 20272 | | *rpoC*2-*rpoC*1 |
| 6 | 34 | F | 55621 | 55964 | | *rbc*L-*trn*R-UCG |
| 7 | 34 | F | 42443 | 141974 | | *ycf*3 intron/*rps*12-*rrn*16 |
| 8 | 34 | F | 55969 | 55978 | | *rbc*L-*trn*R-UCG |
| 9 | 34 | F | 57555 | 57573 | | *rbc*L-*trn*R-UCG |
| 10 | 34 | F | 57572 | 57656 | | *rbc*L-*trn*R-UCG |
| 11 | 35 | F | 57572 | 57665 | | *rbc*L-*trn*R-UCG |
| 12 | 38 | F | 57558 | 57660 | | *rbc*L-*trn*R-UCG |
| 13 | 43 | F | 55758 | 55805 | | *rbc*L-*trn*R-UCG |
| 14 | 44 | F | 55613 | 55622 | | *rbc*L-*trn*R-UCG |
| 15 | 132 | F | 6613 | 6759 | | *trn*Q-UUG-*psb*K |
| 16 | 30 | P | 49533 | 49533 | | *trn*V-UAC-*trn*M-CAU |
| 17 | 31 | P | 74115 | 74115 | | *psb*T-*psb*N |
| 18 | 32 | P | 3095 | 3136 | | *mat*K-*rps*16 |
| 19 | 32 | P | 26980 | 27021 | | *rpo*B-*trn*D-GUC |
| 20 | 33 | P | 55985 | 57669 | | *rbc*L-*trn*R-UCG |
| 21 | 34 | P | 42443 | 98338 | | *ycf*3 intron/*rrn*16-*rps*12 |
| 22 | 34 | P | 55980 | 57563 | | *rbc*L-*trn*R-UCG |
| 23 | 36 | P | 28751 | 28751 | | *psb*M-*pet*N |
| 24 | 42 | P | 31305 | 31352 | | *trn*S-UGA-*psb*C |
| 25 | 52 | P | 80459 | 80459 | | *rps*8-*rpl*14 |
| ***S. yakushimensis*** | | | | | | |
| No. | Size | Type | Start 1 | Start 2 | Location | |
| 1 | 34 | F | 41343 | 152043 | *ycf*3 intron/*rps*12-*rrn*16 | |
| 2 | 31 | F | 47957 | 129802 | *trn*V-UAC intron/*ndh*A intron | |
| 3 | 33 | F | 47959 | 141251 | *trn*V-UAC intron/*chl*L-*trn*N | |
| 4 | 33 | F | 47960 | 47961 | *trn*V-UAC intron | |
| 5 | 33 | F | 92601 | 153198 | *rrn*16-*rps*12 | |
| 6 | 31 | F | 104546 | 104549 | *trn*N-GUU-*chl*L | |
| 7 | 58 | F | 119211 | 120895 | *ycf*1-*ccs*A/*trn*L-*trn*P | |
| 8 | 58 | F | 119221 | 120905 | *ycf*1-*ccs*A/*trn*L-*trn*P | |
| 9 | 37 | F | 119242 | 120926 | *ycf*1-*ccs*A/*trn*L-*trn*P | |
| 10 | 62 | F | 125128 | 125156 | *ndh*F-*ndh*D | |
| 11 | 34 | F | 125128 | 125184 | *ndh*F-*ndh*D | |
| 12 | 36 | F | 129797 | 141246 | *ndh*A intron/*chl*L-*trn*N | |
| 13 | 30 | F | 129803 | 141253 | *ndh*A intron/*chl*L-*trn*N | |
| 14 | 36 | F | 129804 | 141252 | *ndh*A intron/*chl*L-*trn*N | |
| 15 | 31 | F | 129804 | 129807 | *ndh*A intron | |
| 16 | 31 | F | 129807 | 141252 | *ndh*A intron/*chl*L-*trn*N | |
| 17 | 30 | F | 141252 | 141256 | *chl*L-*trn*N | |
| 18 | 63 | P | 2051 | 2051 | *mat*K-*rps*16 | |
| 19 | 38 | P | 27616 | 27616 | *psb*M-*pet*N | |
| 20 | 42 | P | 30201 | 30248 | *trn*S-*psb*C | |
| 21 | 39 | P | 35287 | 35346 | *rps*14-*psa*B | |
| 22 | 50 | P | 36138 | 115460 | *psa*B/*ycf*1-*ccs*A | |
| 23 | 36 | P | 36157 | 115455 | *psa*B/*ycf*1-*ccs*A | |
| 24 | 36 | P | 36189 | 115419 | *psa*B/*ycf*1-*ccs*A | |
| 25 | 34 | P | 41343 | 93755 | *ycf*3 intron/*rps*12-*rrn*16 | |
| 26 | 33 | P | 47959 | 104548 | *trn*V-UAC intron/*trn*N-*chl*L | |
| 27 | 31 | P | 48423 | 48458 | *trn*V-UAC-*trn*M-CAU | |
| 28 | 40 | P | 75388 | 75388 | *rps*8-*rpl*14 | |
| 29 | 33 | P | 92601 | 92601 | *rrn*16-*rps*12 | |
| 30 | 36 | P | 104544 | 129804 | *trn*N-GUU-*chl*L/*ndh*A intron | |
| 31 | 31 | P | 104546 | 129804 | *trn*N-GUU-*chl*L/*ndh*A intron | |
| 32 | 31 | P | 104546 | 141252 | *trn*V-UAC intron/*chl*L-*trn*N | |
| 33 | 30 | P | 104550 | 141254 | *trn*V-UAC intron/*chl*L-*trn*N | |
| 34 | 36 | P | 104550 | 129797 | *trn*N-GUU-*chl*L/*ndh*A intron | |
| 35 | 30 | P | 104550 | 129806 | *trn*N-GUU-*chl*L/*ndh*A intron | |
| 36 | 33 | P | 153198 | 153198 | *rps*12-*rrn*16 | |
| 37 | 30 | R | 47957 | 129804 | *trn*N-GUU-*chl*L/*ndh*A intron | |
| 38 | 36 | R | 47959 | 47959 | *trn*V-UAC intron | |
| 39 | 30 | R | 47959 | 47960 | *trn*V-UAC intron | |
| 40 | 30 | R | 47959 | 141252 | *trn*V-UAC intron/*chl*L-*trn*N | |
| 41 | 32 | R | 47960 | 47960 | *trn*V-UAC intron | |
| 42 | 31 | R | 104546 | 104548 | *trn*N-GUU-*chl*L | |
| 43 | 30 | R | 104550 | 104550 | *trn*N-GUU-*chl*L | |
| 44 | 30 | R | 129802 | 129804 | *ndh*A intron | |
| 45 | 30 | R | 129803 | 141253 | *ndh*A intron/*chl*L-*trn*N | |
| 46 | 32 | R | 129804 | 141252 | *ndh*A intron/*chl*L-*trn*N | |
| 47 | 31 | R | 129804 | 129804 | *ndh*A intron | |
| 48 | 31 | R | 129804 | 141249 | *ndh*A intron/*chl*L-*trn*N | |
| 49 | 31 | R | 129805 | 141253 | *ndh*A intron/*chl*L-*trn*N | |
| 50 | 31 | R | 129805 | 129805 | *ndh*A intron/*chl*L-*trn*N | |
| 51 | 30 | R | 141251 | 141252 | *ndh*A intron/*chl*L-*trn*N | |
| 52 | 31 | R | 141255 | 141255 | *chl*L-*trn*N | |
