# Supplementary figures and images for: Comparative genomic analysis of Polypodiaceae chloroplasts reveals fine structural features and dynamic insertion sequences
Source: BMC Plant Biol. 2021 Jan 7;21:31. doi: 10.1186/s12870-020-02800-x (PMC7792340; doi:10.1186/s12870-020-02800-x)

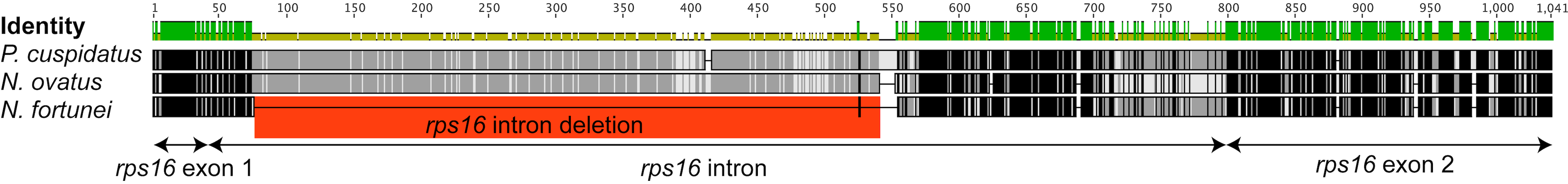

Supplement: Supplementary file 2 — Additional file 2: Figure S1. Alignment of rps16 exons/introns for the three plastomes that we sequenced. The intron is deleted only in N. fortunei. [file 12870_2020_2800_MOESM2_ESM.tif]

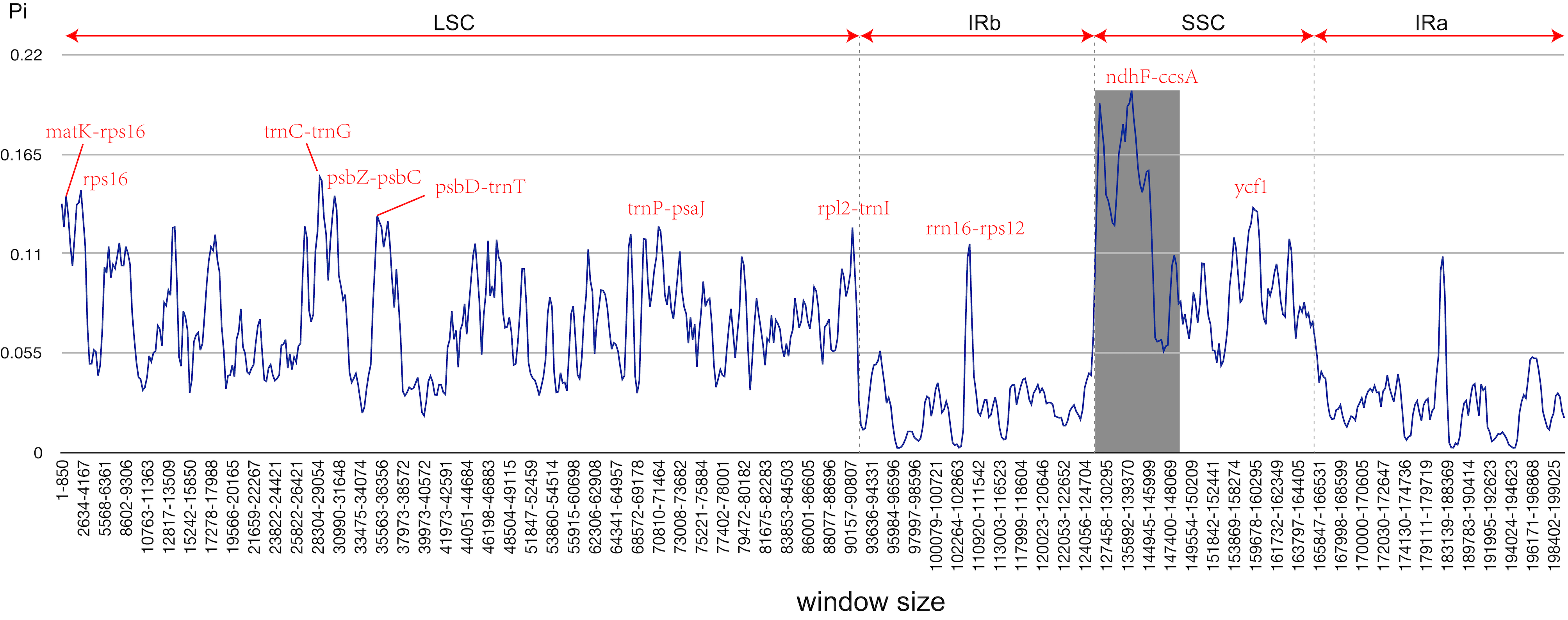

Supplement: Supplementary file 3 — Additional file 3: Figure S2. Nucleotide diversity (Pi) in the plastomes of 12 Polypodiaceae species. [file 12870_2020_2800_MOESM3_ESM.tif]

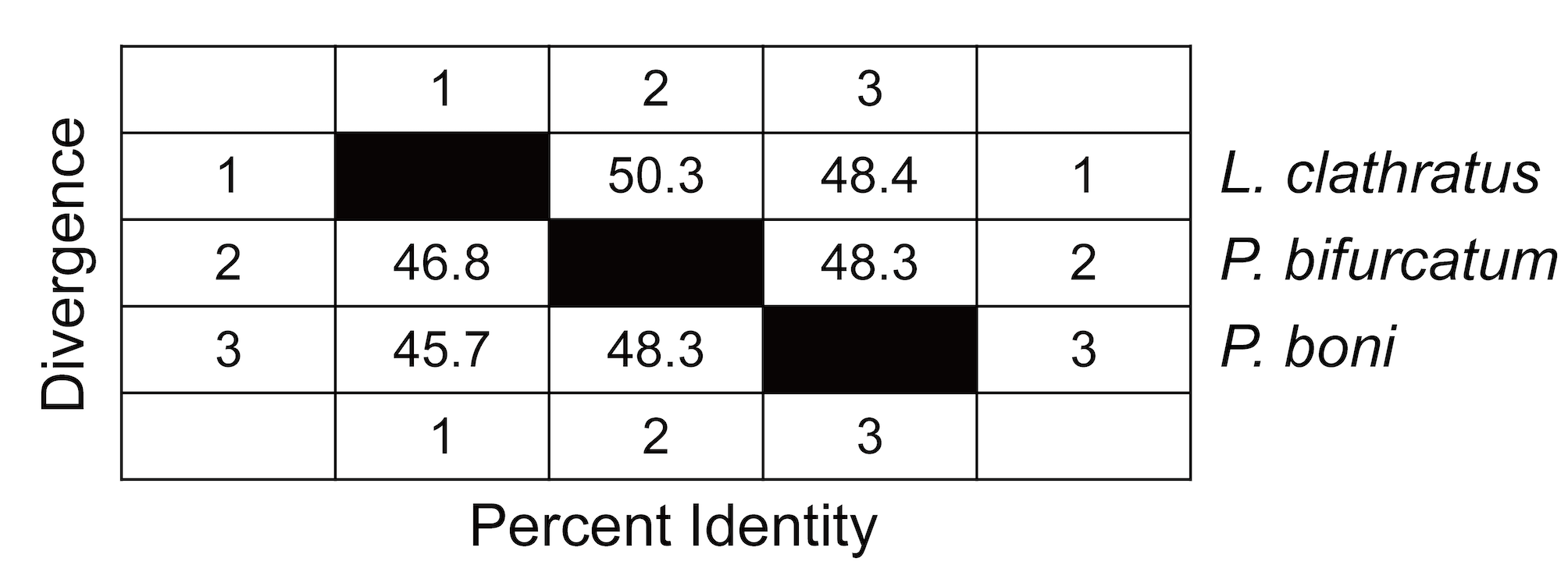

Supplement: Supplementary file 4 — Additional file 4: Figure S3. Identity of large insert fragments in rrn16-rps12 among the plastomes of L. clathratus, P. bifurcatum, and D. roosii. [file 12870_2020_2800_MOESM4_ESM.tif]
